# Development and experimental validation of a machine learning model for the prediction of new antimalarials

**DOI:** 10.1186/s13065-025-01395-4

**Published:** 2025-01-30

**Authors:** Mukul Kore, Dimple Acharya, Lakshya Sharma, Shruthi Sridhar Vembar, Sandeep Sundriyal

**Affiliations:** 1 Department of Pharmacy, Birla Institute of Technology and Science Pilani, Pilani Campus, Vidya Vihar, Pilani, Rajasthan 333 031 India; 2 Institute of Bioinformatics and Applied Biotechnology, Electronics City Phase I, Helix Biotech Park, Bengaluru, Karnataka 560100 India

**Keywords:** Malaria, Machine learning, Random forest, KNIME, Modelling, ChEMBL

## Abstract

A large set of antimalarial molecules (*N* ~ 15k) was employed from ChEMBL to build a robust random forest (RF) model for the prediction of antiplasmodial activity. Rather than depending on high throughput screening (HTS) data, molecules tested at multiple doses against blood stages of *Plasmodium falciparum* were used for model development. The open-access and code-free KNIME platform was used to develop a workflow to train the model on 80% of data (*N* ~ 12k). The hyperparameter values were optimized to achieve the highest predictive accuracy with nine different molecular fingerprints (MFPs), among which Avalon MFPs (referred to as RF-1) provided the best results. RF-1 displayed 91.7% accuracy, 93.5% precision, 88.4% sensitivity and 97.3% area under the Receiver operating characteristic (AUROC) for the remaining 20% test set. The predictive performance of RF-1 was comparable to that of the malaria inhibitor prediction platform (MAIP), a recently reported consensus model based on a large proprietary dataset. However, hits obtained from RF-1 and MAIP from a commercial library did not overlap, suggesting that these two models are complementary. Finally, RF-1 was used to screen small molecules under clinical investigations for repurposing. Six molecules were purchased, out of which two human kinase inhibitors were identified to have single-digit micromolar antiplasmodial activity. One of the hits (compound **1**) was a potent inhibitor of *β*-hematin, suggesting the involvement of parasite hemozoin (Hz) synthesis in the parasiticidal effect. The training and test sets are provided as supplementary information, allowing others to reproduce this work.

**Supplementary Information:**

The online version contains supplementary material available at 10.1186/s13065-025-01395-4.

## Introduction

Malaria is an infectious disease caused by *Plasmodium*, a protozoan parasite, and transmitted by the bite of a female Anopheles mosquito. As per the World Health Organization’s (WHO) 2023 World Malaria Report, globally, there were ~ 249 million malaria cases in 2022, of which 609,000 were fatal [1]; about 94% of these cases were estimated to occur in the WHO African Region. Although two new vaccines are now available for malaria prevention in children [1], malaria therapy using small molecules is facing tremendous challenges. The *Plasmodium* parasite has developed resistance to all clinically available drugs leading to increased incidences of multi-drug resistant (MDR) malaria [2, 3]. Artemisinin-based combination therapies (ACTs) are prescribed to tackle MDR malaria [4], however, treatment failures with ACTs are increasingly being reported [5, 6]. Thus, novel small molecules with unique mechanisms of action are needed to circumvent the resistance problem and improve clinical outcomes.

Machine learning (ML) and artificial intelligence (AI) methods are increasingly being applied in drug discovery [7–11]. One of the major applications of ML and AI is the development of predictive models to estimate a compound’s biological activity, toxicity, or physicochemical properties. The availability of such predictive models can reduce the overall cost of the drug discovery process leading to affordable drugs. ML-based screening of approved drugs or investigational agents can also assist in repurposing of molecules with acceptable pharmacokinetics and safety profile [12]. The low cost of newly discovered antimalarials is an important criterion set by WHO given the prevalence of malaria in low-income Sub-Saharan countries [13].

In the past 10–15 years, several high throughput screening (HTS) campaigns have been carried out to identify antimalarial small molecules with new chemotypes and novel modes of action [14–22]. This valuable data can be used to understand the antimalarial chemical space [23, 24] and build predictive models to screen for as-yet-unknown antimalarial chemotypes. For instance, Jamal et al. developed ML models for the prediction of molecules that inhibit the parasite’s apicoplast function leading to delayed death phenotype [25]. However, the authors used a highly imbalanced dataset of ~ 323 K compounds deposited in PubChem for model building, out of which only ~ 22 K were classified as “actives”. Indeed, their best model based on random forest (RF) yielded an area under the Receiver operating characteristic (AUROC) curve of only 70%. Danishuddin et al. employed a dataset of 4750 molecules obtained from the ChEMBL database to develop predictive ML models of parasite killing activity against asexual blood-stages (ABS) using Support Vector Machine (SVM), k-nearest neighbours (k-NN), RF, and XGBoost [26]. The models were validated using an external dataset reported in PubChem, achieving an AUROC curve of approximately 85%. Mughal et al. developed a RF model to predict new molecules that could be active against parasite liver stages, without being cytotoxic to mammalian cells [27]; their training set consisted of 5972 compounds found to be active in an HTS study against *Plasmodium berghei* culture in human hepatoma HepG2 cells. Heerden et al. used HTS data to build SVM models for predicting individual or dual activity against ABS and sexual blood stages of *P. falciparum* [28]. Lastly, Bosc et al. created a large HTS dataset of antiplasmodial compounds obtained from different organizations and used this dataset to develop ML models [29–31]. Since a majority of the compounds were proprietary, a unique approach was adopted to develop a consensus model without sharing compound information: an ML model was built at each organization with proprietary datasets, following which a metamodel was generated using the weighted Morgan fingerprint (MFP) bits from individual models. The resulting Naïve Bayes (NB)-based MAlaria Inhibitor Prediction (MAIP) model was also experimentally validated [31]. 

Most of the earlier reported ML models for antimalarial activity are based on results from phenotypic HTS screening. This means that the compounds are tested at a single dose which only indicates whether a compound is active or inactive at that dose. This binary result does not reveal the compound’s potency or efficacy and there is high probability of compound being a false positive or false negative. Such data is expected to be detrimental for the accuracy of the model as per the garbage in, garbage out principle. Moreover, the HTS datasets used in previous studies were small and unbalanced, which may have impacted model optimization and validation. To overcome these challenges, we decided to use input molecules that are evaluated at varying doses. A compound displaying a biological end-point in a dose-dependent manner is more reliable. A truly active molecule displays a typical dose-response curve from which IC_50_/EC_50_ values can be deducted which are constant under the given assay conditions. Fortunately, a large dataset of antiplasmodial molecules with reported IC5_0_/EC_50_ is available in ChEMBL database [32]. The latter is publicly available database which is updated regularly from the literature. Thus, to generate a reliable and robust ML model we selected a large and balanced dataset of antiplasmodial molecules tested at multiple doses against *P. falciparum* ABSs. The optimization of the model, its comparison with existing MAIP model, and experimental validation is also provided.

## Results and discussion

### Data curation and preprocessing

Our objective was to develop a classification model that could differentiate between “active” and “inactive” classes of compounds when tested in a typical phenotypic antiplasmodial assay. The robustness of any computational model is strongly related to the quality of input data. The more reliable the data used for training the model, the better the model’s performance. Therefore, for our model, we used a dataset of compounds with reported IC_50_/EC_50_ values, i.e., molecules that were tested at multiple doses and showed a dose-dependent parasite-killing phenotype. We recently compiled such a dataset for the physicochemical profiling and chemical space characterization of antimalarial compounds [23, 24]. Our dataset contains ~ 15,000 molecules tested against the ABS of malarial parasites and was curated from the ChEMBL database [32, 33], one of the largest databases of bioactive chemical compounds curated from reputed peer-reviewed medicinal chemistry literature.

**Fig. 1:**
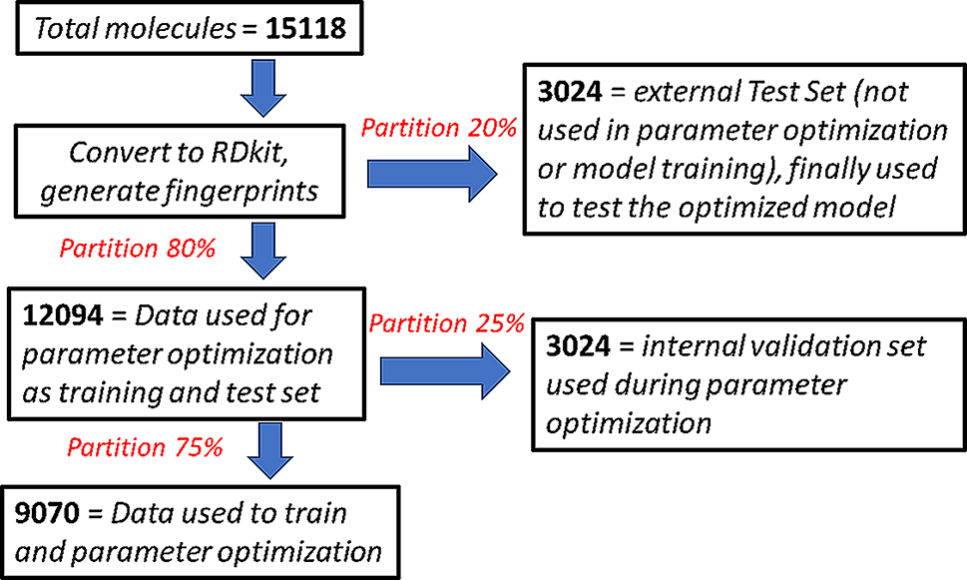
Data curation and partitioning of the molecules into training, internal validation, and external test sets. As per the standard practice, the external test set is used only for the validation of the optimized model

Within our dataset, we defined “actives” as having IC_50_ < 200 nM (*N* = 7039) and “inactives” as having IC_50_ > 5000 nM (*N* = 8079). Compounds with intermediate activity were not considered to have a clear demarcation between the two classes and noise-free input. 20% (*N* = 3024) of the total dataset (*N* = 15118) was kept aside as the external “test set” and was later used to evaluate the predictive performance of the final optimized model. The remaining set of molecules (*N* = 12094) were further partitioned into training (75%, *N* = 9070) and internal validation set (25% *N* = 3024). The latter was used during parameter optimization (Fig. 1).

### Model development and hyperparameter optimization

Several ML algorithms such as Decision Tree (DT), Artificial Neural Network (ANN), SVM, k-NN, RF, and NB, are available for classification and regression modelling. Although ANN based modern methods are also popular for predictive modeling, these are often prone to overfitting and computationally expensive. Also, ANN methods lack interpretability and considered as ‘black box’ models. Among these methods we selected RF technique owing to its higher speed and robustness against overfitting [34, 35]. Also, RF has found several applications in medicinal chemistry in the past and consistently outcompetes other algorithms in terms of prediction accuracy and robustness [35–41]. RF benefits from the ‘wisdom of the crowd’ effect as it is based on an ensemble of independent decision trees (DTs) which contribute to the overall prediction. The RF algorithm involves the ‘bagging’ of data in which a random sample of the training set is selected for training individual DTs. The data sample is selected with replacement (bootstrapping), meaning that a data point may be part of more than one random sample [34, 35]. Thus, after each random sampling of the training set, typically one-third of the data points are left out which are referred to as out-of-bag (OOB) samples. During the individual tree construction, the OOB samples are used as the validation set since these are not used during model training. Consequently, the prediction accuracy of the OOB sets is an important internal cross-validation to assess an RF model. Another important aspect of RF is that it employs only a random subset of features or attributes (independent variables such as descriptors, MFP bits, etc.) for training a tree which is typically the square root of the total number of features. This makes the RF algorithm much faster than DTs, especially when a large number of variables are used, since only a subset of attributes is tested for their splitting performance at each node [35].

**Fig. 2:**
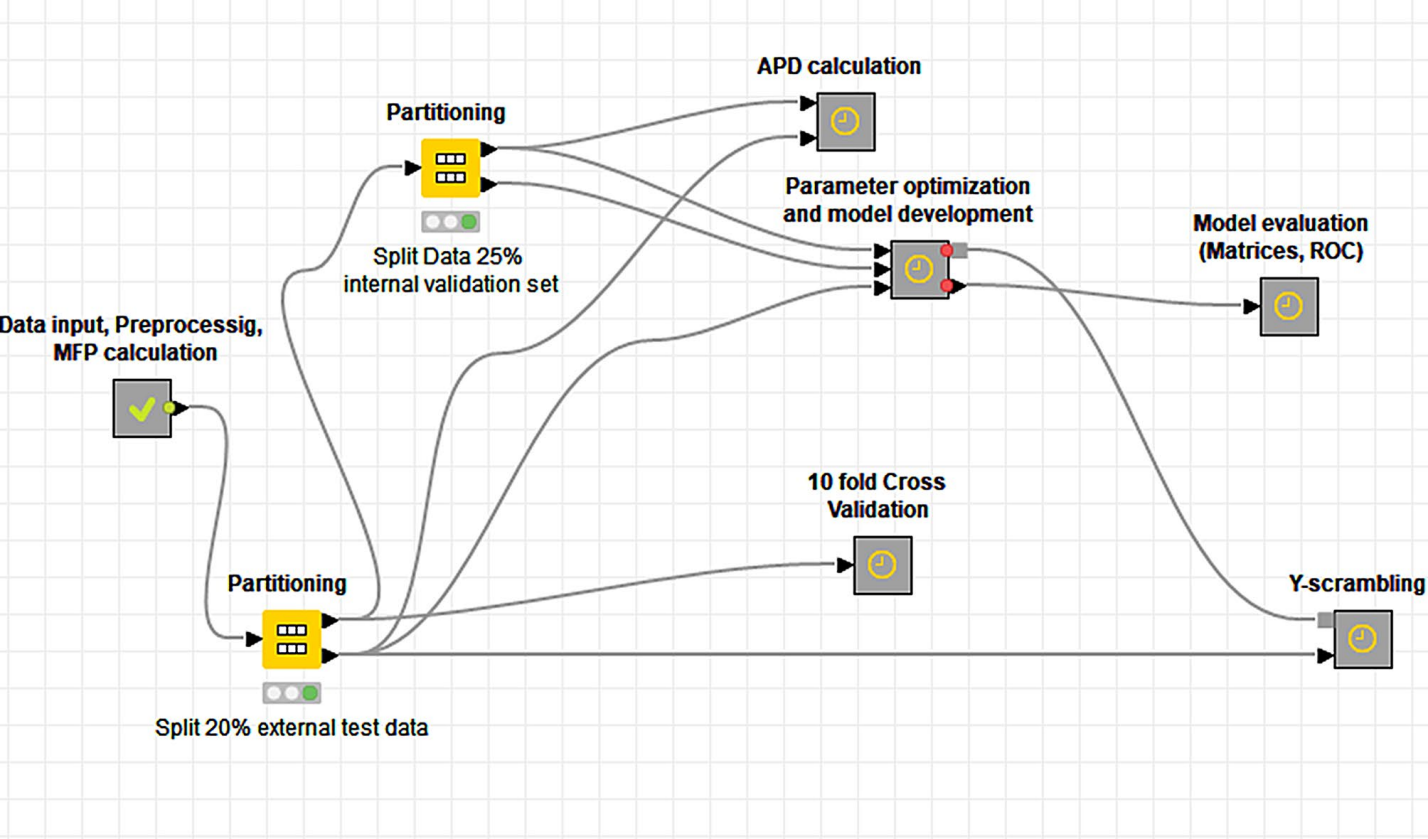
Overview of the KNIME workflow employed for RF model development, optimization, and validation. The yellow box represents a node capable of carrying out a specific task. The grey boxes depict a metanode consisting of several nodes (yellow box) connected to perform multiple tasks. Each metanode is a group of more than one nodes contributing to the specific task of the metanode

To build our model, we employed the Konstanz Information Miner (KNIME) platform, a versatile, freely available data analytics platform [42–44]. In KNIME, nodes can be combined to develop a workflow to automate a variety of tasks including data curation, visualization, and machine learning modeling. Several open-source nodes are available to manipulate chemical structures and to calculate a variety of MFPs and chemical descriptors [45]. Since RDkit is the widely used open-source cheminformatics software [46] we used it for MFP calculations in KNIME. Each MFP represents the chemical structures in different ways and have their own advantages and disadvantages [47]. Thus, the best performing MFP should be determined for a specific objective during model optimization. Next, we used the ‘RF Learner’ and ‘RF Prediction’ nodes available in KNIME to build a predictive model. Since the nature of MFPs might affect the model accuracy, we optimized the model to select the best-performing MFP. The number of DTs to be learned (nT) and tree depth (Td, or number of levels) are important factors that may affect the overall performance of the RF model and the amount of computational time. Employing higher nT and Td values requires more time for model construction but may not lead to higher accuracy [35]. Therefore, the values for nT and Td hyperparameters need to be optimized during model optimization. Using the KNIME loop nodes, we varied the model nT and Td values between 50 and 400 and 10–50, respectively, and recorded model accuracy for each combination. Thus, we obtained optimized values for the hyperparameters for each MFP employing the training set (*N* = 9070) and internal validation set (*N* = 3024). Finally, the optimized nT and Td values were used to evaluate the models against the external test set (*N* = 3024). The latter set of molecules was neither used for training nor for optimizing the model as per standard practice. The overall workflow is depicted in Fig. 2.

The results showed that most of the MFPs performed well displaying over 90% accuracy for the optimized models (RF-1-9) except for the atom pair MFP (Model 9, Table 1). Among the top performers, models based on Avalon, (RF-1), Feature Morgan (Model 2), and Layered (Model 3) MFPs displayed very similar accuracies. However, Avalon FPs displayed the highest accuracy (91.7%), with the lowest optimized values of 150 and 50 for nT and Td, respectively. Thus, optimized RF-1 is found to be best in terms of overall accuracy and speed. However, accuracy alone can be misleading, and other metrics are prescribed for evaluating different aspects of the model depending on the objective. These metrics possess values between 0 and 1, with higher values representing the better-performing model. All these metrics can be calculated from the confusion matrix (Figure S1, Supplementary information file 1) that represents the number of true positives (TPs), true negatives (TNs), false positives (FPs), and false negatives (FNs).

The overall accuracy of a machine learning model represents the percentage of data points predicted correctly (TP + TN) by the model. Precision represents the quality of positive predictions by the model and refers to the proportion of TP cases (in this case active molecules with IC_50_ < 200 nM) out of all predicted positive cases (TP + FP). The sensitivity (or recall) is the true positive rate (TPR) and is calculated as the proportion of TP cases of actual positive cases (TP + FN). The specificity is the proportion of actual negative cases (TN) that are correctly identified out of the total negative cases predicted by the model (TN + FP). In addition to the overall highest accuracy, RF-1 displays the highest values for sensitivity (0.884). One of the matrices for evaluating the balance between precision and sensitivity is the F1 measure or score. The latter is simply a harmonic means of precision and sensitivity. RF-1 displayed the highest F1-score (0.908) suggesting it to be a good predictor for both positive (active) as well as negative classes (inactive). The use of F1-score matric is relevant in this study as the test set is balanced, that is, molecules in both active (*N* = 1408) and inactive classes (*N* = 1616) are almost equal.

**Table 1:** Optimized RF models based on different fingerprints and corresponding matrices for the external test set

Model	MFP	nT, Td	Accuracy	Precision	Sensitivity (recall)	Specificity	MCC	Cohen’s kappa	F-measure	AUROC
RF-1	Avalon	150, 50	0.917	0.935	0.884	0.946	0.834	0.833	0.908	0.973
RF-2	Feature Morgan	200, 50	0.916	0.951	0.864	0.961	0.833	0.830	0.905	0.971
RF-3	Layered	200, 20	0.915	0.934	0.879	0.946	0.829	0.828	0.906	0.969
RF-4	RDKit	350, 20	0.912	0.948	0.859	0.959	0.826	0.823	0.901	0.969
RF-5	Morgan	100, 50	0.911	0.951	0.853	0.962	0.824	0.820	0.899	0.970
RF- 6	Torsion	400, 40	0.909	0.941	0.859	0.953	0.819	0.816	0.898	0.969
RF-7	Pattern	250, 40	0.908	0.928	0.870	0.941	0.816	0.815	0.898	0.967
RF-8	MACCS	150, 30	0.905	0.912	0.881	0.926	0.809	0.809	0.896	0.963
RF-9	Atom Pair	250, 40	0.896	0.912	0.859	0.928	0.791	0.790	0.885	0.956

Cohen’s Kappa (κ) [48] represents the agreement between two raters, in this case, actual and predicted values by the model. The value of κ lies between − 1 to + 1, the more positive value represents the better predictive performance of the model in comparison to random classification. Among all, RF-1 showed the highest value for the κ (0.833) representing substantial agreement between the actual and the predicted values. Matthews correlation coefficient (MCC) is another metric for model evaluation [49, 50] that shows advantages over F1 and accuracy, especially in the case of imbalanced data [51]. Again RF-1 displayed the highest MCC (0.834) among all supporting its reliability and accuracy.

**Fig. 3:**
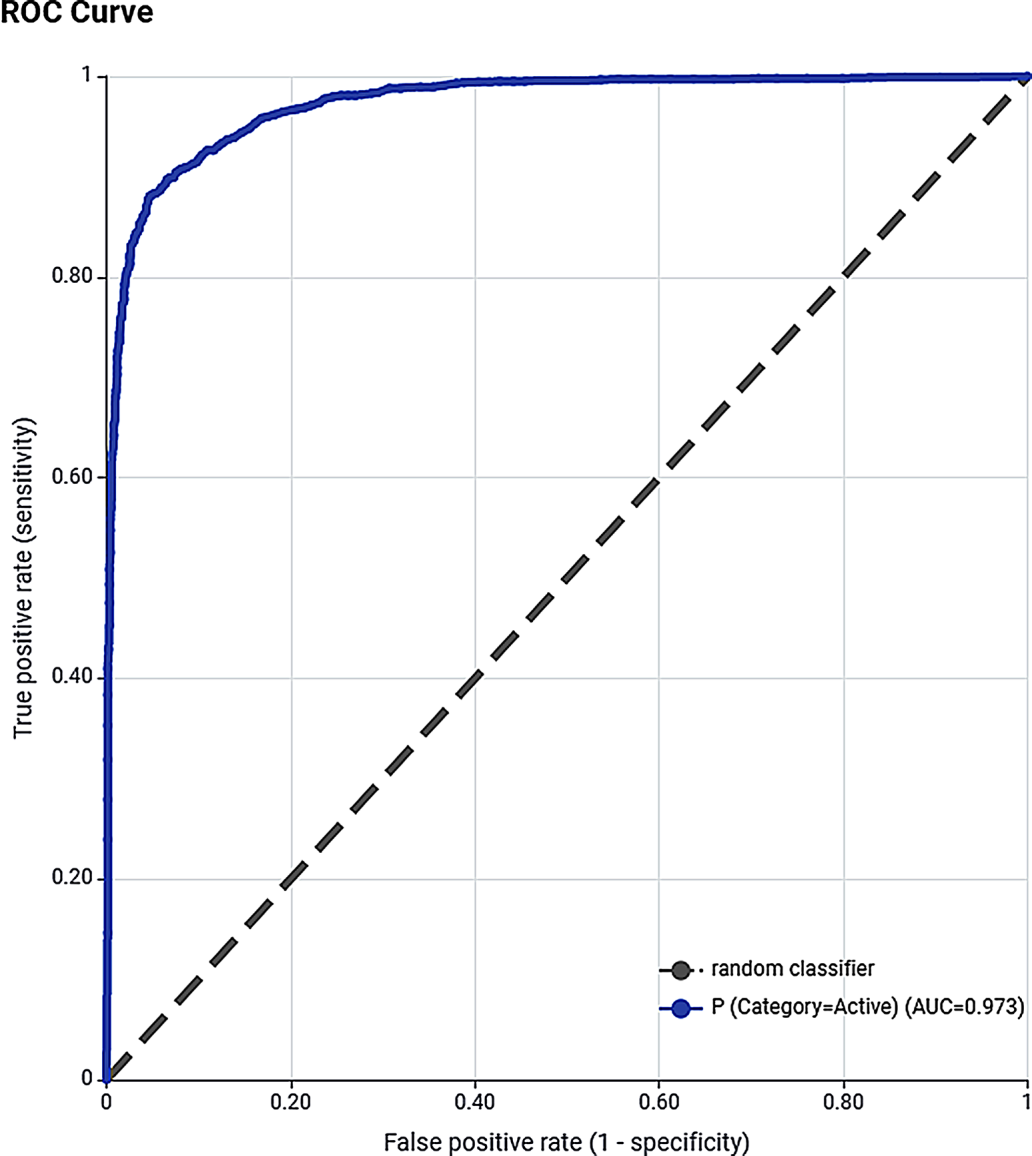
ROC curve obtained with RF-1. The diagonal represents a random classifier with 50% AUROC while RF-1 displays 97.3% AUROC for the external test set showing the high discriminatory power of the model for active and inactive antimalarial compounds

Another crucial criterion for evaluating a two-class classification model is the ROC curve. The ROC is a graphical plot between Sensitivity (TPR) and false positive rate (FPR or 1 –Specificity) at varying probability thresholds. Thus, the ROC curve extracts information from multiple confusion matrices by varying the threshold to discriminate between the two classes (active vs. inactive). A two-class classifier model with no discriminative power would show as a diagonal, that is, point (0.5, 0.5) at all thresholds. In contrast, an ideal model is represented at a point (0,1), travelling from the bottom left to the top left and then across the top to the top right, thus displaying the maximum value for AUROC. The AUROC matrix can be used to compare different classifier models’ predictive capacity. A completely ineffective 2-class model will have AUROC of 0.5 (or 50%) probability while a perfect model will display a value of 1 (or 100%) AUROC. RF-1 displayed the highest AUROC of 0.973 (Fig. 3) suggesting it to be an excellent discriminator between the two classes and can be employed to predict novel antimalarial molecules.

### Model validation

#### OOB accuracy

**Fig. 4:**
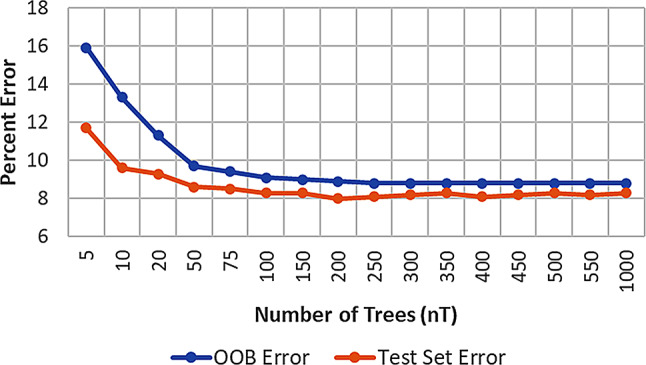
The variation of OOB and the external test set error rates show similar trends with the increasing number of trees. This validates the consistency of models in calculating error rates for the training set and test set

On average, during RF modeling, each tree is constructed using two-thirds of the rows in the training set and the remaining one-third of samples are predicted by the RF model. This constitutes an internal validation of the model yielding an OOB confusion matrix. Each row of the training set is predicted by the majority vote of all the other trees that did not use the row during their training. Therefore, OOB accuracy is different from the accuracy of the model observed for the test set and allows the training of a validated model. As expected, both OOB and test error rates tracked well [35] and displayed almost constant values after the usage of ~ 100 trees (Fig. 4). This agrees with the known fact that increasing the number of trees above the optimum value doesn’t improve model accuracy. Also, this observation is in congruence with the optimized nT value obtained after the parameter optimization using Avalon MFP (RF-1).

The accuracy and other matrices obtained from OOB confusion matrix (Table S1, Supplementary information file 1) are also close to the finally optimized RF-1 (Table 1) suggesting RF-1 to have high predictive performance.

#### 10-fold cross-validation (CV)

We performed a 10-fold CV of RF-1 (nT = 150, Td = 50, Avalon MFP) employing the complete dataset (*N* = 12094) except for the external test set. This approach involves randomly dividing the dataset into 10 folds of equal size. Each time the model is trained with 9 folds, and the 10th fold is used as the validation set. Thus, a total of 10 models are trained and evaluated on the hold-out validation sets. The 10-fold CV was repeated ten times with a different random seed to ensure different combinations of the folds. The mean and standard deviations of accuracy and other evaluation matrices were found to be very similar to each other in all repeats (Table 2) demonstrating the stability of RF-1. In addition, after multiple iterations average CV error rate is expected to converge to the OOB error rate [35] which was found to be true with RF-1. The other evaluation matrices from CV are almost identical to the values obtained for the test set further providing the evidence of robustness and predictive performance of RF-1.

**Table 2:** Results obtained from 10-fold CV repeated ten times

Seed	Accuracy	Precision	Sensitivity (Recall)	Specificity	Cohen’s kappa	F-measure	AUROC
0	0.911 ± 0.008	0.938 ± 0.008	0.880 ± 0.016	0.949 ± 0.007	0.833 ± 0.017	0.908 ± 0.010	0.971 ± 0.005
1	0.915 ± 0.007	0.936 ± 0.012	0.877 ± 0.016	0.948 ± 0.011	0.828 ± 0.014	0.906 ± 0.008	0.971 ± 0.003
2	0.916 ± 0.010	0.936 ± 0.006	0.879 ± 0.023	0.948 ± 0.006	0.828 ± 0.014	0.907 ± 0.012	0.970 ± 0.004
3	0.912 ± 0.010	0.933 ± 0.016	0.879 ± 0.023	0.945 ± 0.014	0.823 ± 0.021	0.903 ± 0.011	0.970 ± 0.007
4	0.917 ± 0.007	0.937 ± 0.012	0.882 ± 0.010	0.948 ± 0.011	0.834 ± 0.014	0.909 ± 0.008	0.971 ± 0.003
5	0.917 ± 0.008	0.937 ± 0.014	0.881 ± 0.011	0.948 ± 0.013	0.833 ± 0.015	0.908 ± 0.008	0.970 ± 0.004
6	0.917 ± 0.008	0.936 ± 0.011	0.882 ± 0.019	0.947 ± 0.010	0.832 ± 0.017	0.908 ± 0.010	0.971 ± 0.004
7	0.916 ± 0.006	0.936 ± 0.008	0.880 ± 0.014	0.947 ± 0.008	0.830 ± 0.013	0.907 ± 0.007	0.970 ± 0.005
8	0.917 ± 0.006	0.937 ± 0.009	0.881 ± 0.011	0.948 ± 0.008	0.832 ± 0.011	0.908 ± 0.006	0.971 ± 0.003
9	0.914 ± 0.005	0.935 ± 0.006	0.877 ± 0.010	0.947 ± 0.005	0.827 ± 0.011	0.905 ± 0.006	0.971 ± 0.004

#### Y-scrambling

Y-Scrambling (Target Shuffling or Y-Randomization) is a method to ensure that the predictions made by the model are not obtained by chance. In this approach, target column values of the test set are shuffled, and the trained model is used to compute the accuracy. If there is no true correlation between the target column and the attributes (or descriptors) then the predictions of Y-shuffled data will still be predicted accurately. Thus, for a binary classification model values for the accuracy and other matrices should be close to 0.5 for the Y-shuffled test data set resembling the prediction by chance. Indeed, RF-1 displayed an accuracy of only 0.508 (Table 3) for the Y-scrambled test set, in contrast to 0.917 for the non-shuffled test data. This confirms that RF-1 is not obtained by chance and the antiplasmodial activity of the molecules is indeed dependent on the MFP attributes used to develop and optimize the model.

**Table 3:** Model performance matrices after Y-scrambling. The values after shuffling the data fall close to 0.5 suggesting a real correlation between the target class and attributes

Parameter	Value
Accuracy	0.508
Sensitivity (recall)	0.444
Precision	0.470
Specificity	0.563
F-measure	0.456
AUROC	0.492

### Applicability domain (APD)

ML models are trained on finite datasets and may not generalize well to data far from the training set. For instance, the model developed using a set of small molecules cannot be used to predict the activity/properties of peptide like molecules. Such an application of ML model will be unreliable due to different chemical spaces of training and prediction set of molecules. Therefore, for all predictive models, an APD threshold is defined, and the distance of each test compound (or virtual screening set) is determined and compared to its nearest neighbour in the training set [52–54]. If the similarity is beyond the predefined APD threshold, the predictions are flagged as unreliable.

**Fig. 5:**
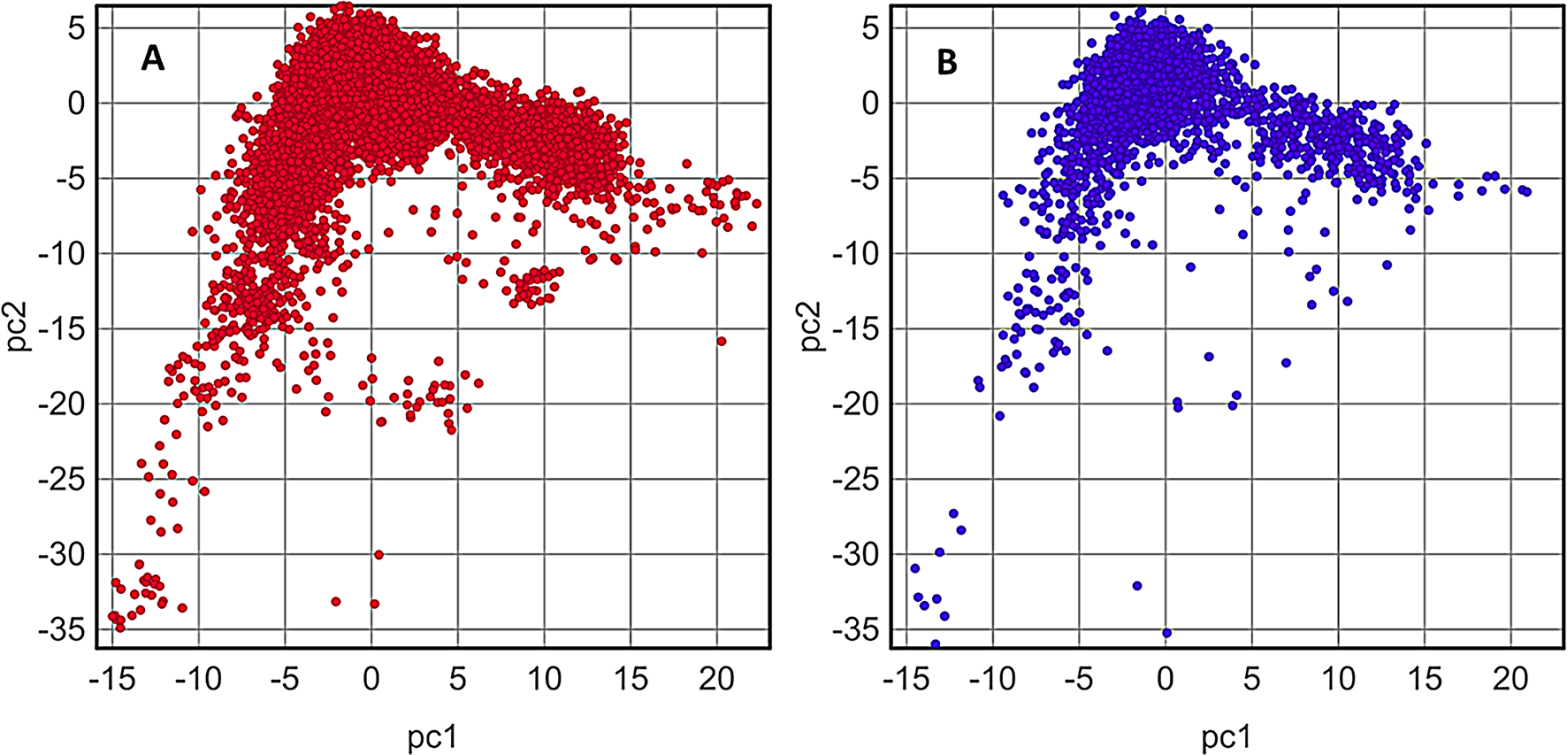
The PCA analysis of (**A**) training and (**B**) test set molecules using the Skelsphere descriptor. The plots display that the training and test molecules occupy a similar chemical space and hence RF-1 can be used reliably to classify the test molecules

Many methods and their comparative studies are available in literature for defining APD thresholds [55–57]. For RF-1, we determined APD using the Euclidean distances between training and test molecules [54]. The 1024-bit vector descriptor space obtained from the Avalon MFPs was used to find out the Euclidean distances as a measure of similarity between all the pairs of training sets. For the test set only 12 out of 3024 (0.4%) of molecules were found to be predicted unreliably using the Avalon descriptors, suggesting test molecules to be within the applicability domain of the RF-1. In addition, principal component analysis (PCA) analysis of the training and test molecules was performed using the structural Skelsphere descriptors [58] available in Datawarrior. The molecules of both sets were found to be within the same boundaries of the PC1 and PC2 axis (Fig. 5) signifying the chemical space similarity of the two sets.

### Comparison of RF-1 with the MAIP platform

The performance of newly developed models is often compared with existing models. Such comparisons may show similarities and differences in terms of hit selection by the models. Previously, we have characterized property space of reported antimalarials [23, 24]. We also demonstrated that MAIP model selects hit molecules conforming to this space, thus providing indirect validation. In addition, MAIP is one of the most recent antimalarial models and is based on a large and diverse dataset (> 6.5 million compounds). Therefore, we set to compare our model with MAIP especially in terms of the property space of the hit molecules. Although MAIP model is based on the proprietary and undisclosed training set it is freely available as a web service platform [29, 31] enabling comparison with our model. The MAIP adopts a consensus approach where Naïve Bayes ML models developed by individual organizations on their datasets are combined to determine the final prediction of the antimalarial activity. However, unlike RF-1, the MAIP model is based on the HTS results and not the IC_50_ values. Consequently, the criteria for the classification of actives and inactive varies for MAIP and RF-1, the latter being based on IC_50_ values rather than percentage inhibition at a single dose.

**Fig. 6:**
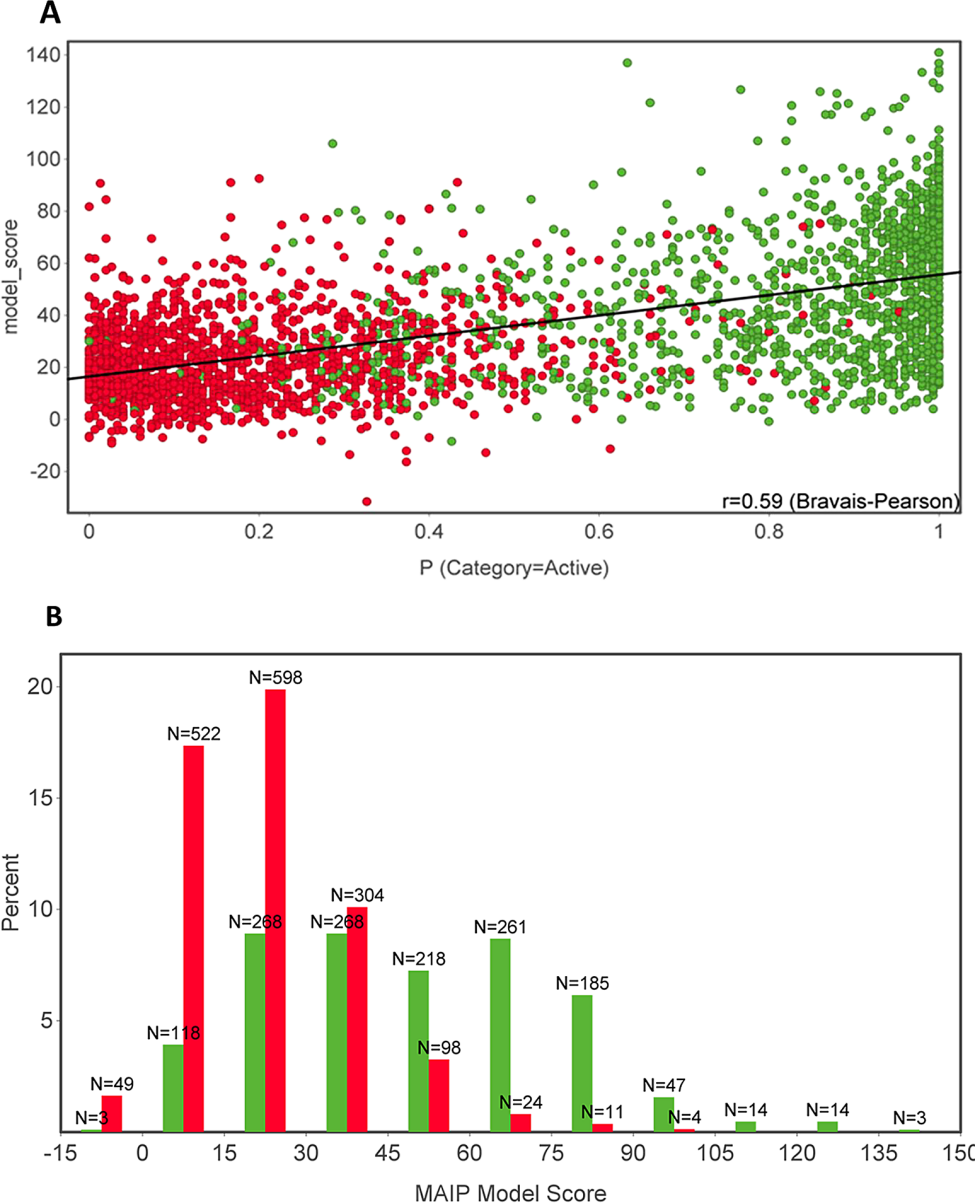
The comparison of the MAIP model and RF-1 against the external test set. The true active molecules are rendered as green circles while true inactive ones as red circles. **A**) correlation of MAIP model score and probability values of active class obtained from RF-1. **B**) The distribution of the MAIP model score for the external test molecules. The true active molecules are also assigned a higher score by the MAIP model suggesting the agreement between the two models

We screened our external test set compounds (*N* = 3024) using the MAIP platform, which provided a model score for each compound rather than class prediction. A higher MAIP model score signifies stronger chances of a compound being a true positive, i.e., active in cell-based antiplasmodial assays. Although the predictive performance of RF-1 and MAIP cannot be compared directly, the MAIP model score was compared with the probability of class prediction obtained from RF-1. The MAIP model score displayed a positive correlation (*r* = 0.590) with RF-1 “active” class probabilities, suggesting congruence between the two models (Fig. 6A). MAIP model score above 45, representing ~ 29% of the test set, showed significant enrichment with active molecules (Fig. 6B).

**Fig. 7:**
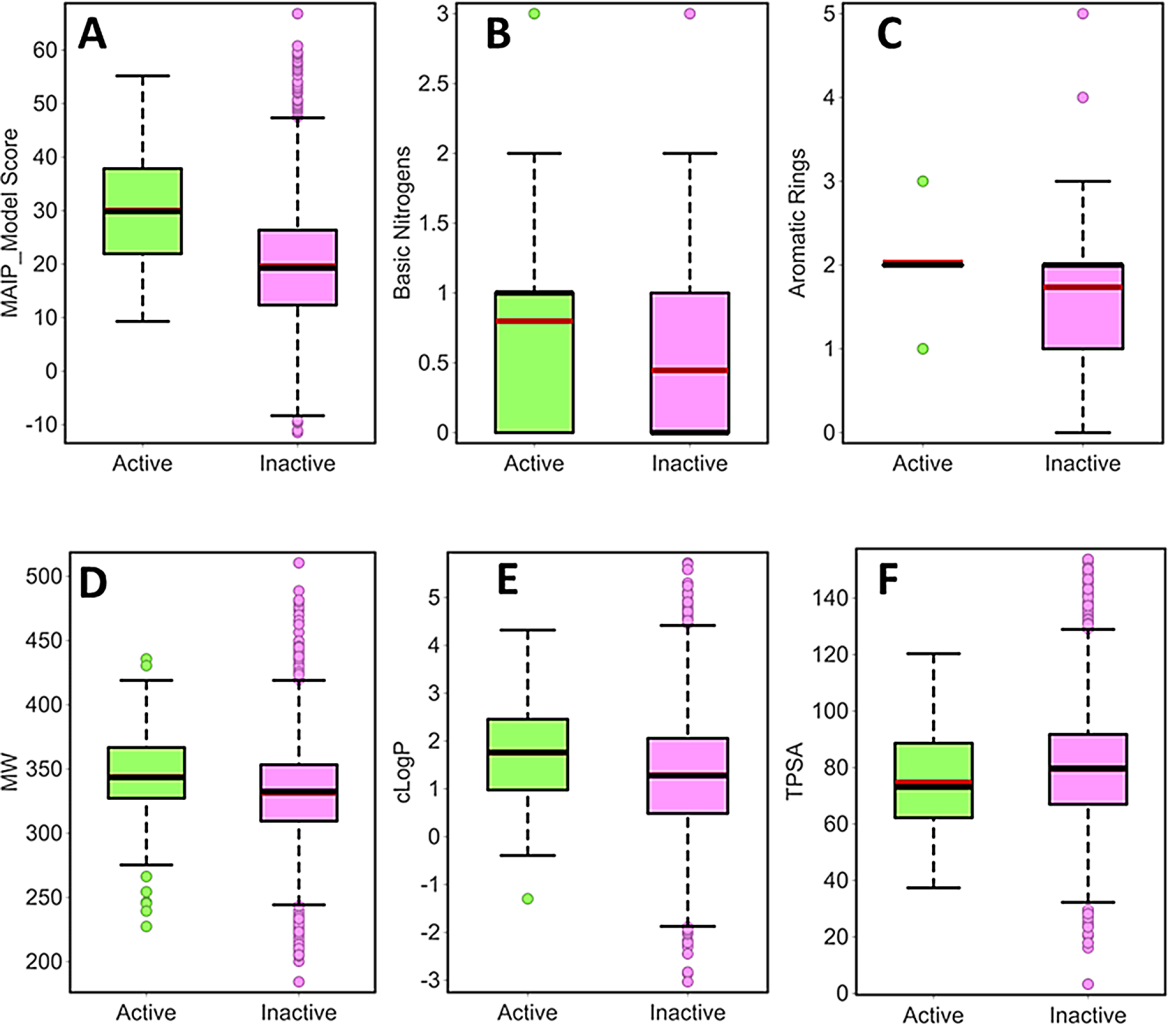
The box plots comparing average values for **A**) MAIP model score, **B**) #BaN, **C**) #AR, **D**) MW, **E**) clogP, and **F**) TPSA, of the predicted active and inactive compounds from the Enamine diversity library. The molecules predicted to be active by RF-1 also show higher MAIP score, on average. The active molecules also conform to antimalarial property space

For further comparison, we screened a diverse set of ~ 10,000 commercially available compounds from Enamine vendor using MAIP and RF-1. As expected, the MAIP scores of 129 hits obtained from RF-1 were higher (average score ~ 30.0) than the remaining molecules (average score 19.6, Fig. 7A). The same library yielded 128 hits with a significant MAIP model score (≥ 45). Interestingly, the hits predicted by MAIP and RF-1 did not overlap. Out of the 129 hits obtained by RF-1, only 30 displayed Skelsphere similarity of 70% or higher to at least one of the MAIP model hits (Figure S2, Supplementary information file 1). Only nine molecules were found common to both hit lists (Figure S3, Supplementary information file 1) and are strong candidates for future experimental validation. Four of these molecules consist of quinolines and quinazolines rings, important antimalarial pharmacophores [24, 59–61]. These chemotypes are still popular [23] for antimalarial drug design and are often recycled with novel substituents [62] against drug-resistant parasites.

Earlier, we proposed an antimalarial property space after profiling a large dataset of research stage, clinical candidates, and marketed antimalarials [23, 24]. We showed that, compared to other oral drugs, antimalarials possess higher molecular weight (MW), calculated partition coefficient (clogP), basic nitrogen count (#BaN), and aromatic ring counts (#AR). In contrast, antimalarials have lower topological surface area (TPSA). Indeed, the hits obtained from phenotypic screens and the MAIP model adhere to this property space [24, 31]. In agreement, the hits predicted by RF-1 also display significantly higher averages for #BaN, #AR, clogP, and MW, while a lower average for TPSA (Fig. 7B − 7 F; Table S2, Supplementary information file 1).

Overall, these results suggest that both MAIP and RF-1 provide hits that occupy the antimalarial property space. However, both models proposed different chemotypes as hits from a diverse library which may be due to the different size and different diversity of the training sets. Also, the model building approach and the definition of active and inactive molecules are different for both models. Therefore, we suggest that both models can be used in synergy for predicting novel antiplasmodial molecules.

### Experimental validation with investigational agents

Repurposing of existing drugs that are proven to be safe and bioavailable is an important strategy in drug discovery [12]. Extensive repurposing study of FDA-approved drugs for antimalarial discovery has been reported earlier [63–66]. Therefore, for repurposing studies with our ML model, we focused on the compounds being investigated in clinical trials. To this end, we obtained a library of investigational compounds from Drugbank database [67]. Metallic, inorganic, and gaseous molecules were removed and the library was filtered to keep compounds with molecular weight between the range of 200–900 Da to fit into the antimalarial property space [23, 24]. The resulting set of 3308 investigational compounds were screened using RF-1, out of which 153 hits were predicted to be active within the applicability domain of the model. For the sake of novelty, compounds with high similarity (≥ 0.8) with the training and test members were eliminated to limit the hit list to 94 compounds. Finally, based on diversity, commercial availability, and cost, we purchased six molecules for antiplasmodial screening (Table S3; Supplementary information file 1).

**Fig. 8:**
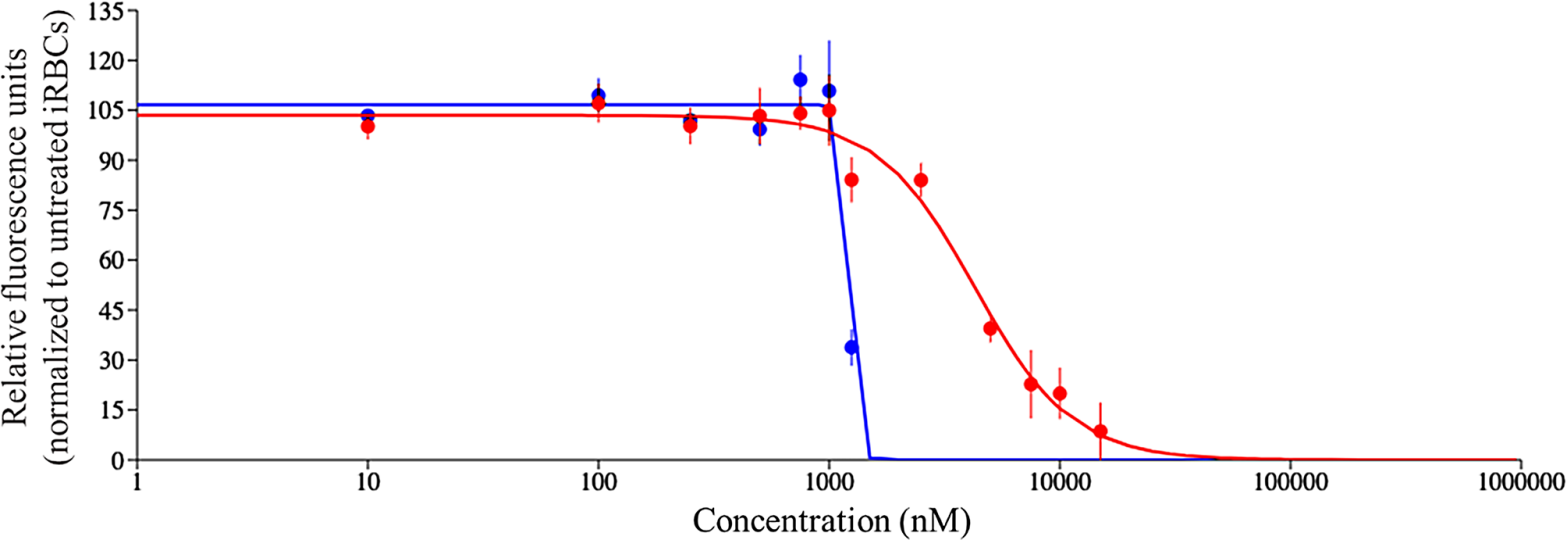
The dose response curves for compounds **1** (blue curve) and **2** (red curve)

The molecules were tested in vitro for anti-plasmodial activity in the SYBR green I assays, which is a standard assay used to evaluate the parasite growth inhibition [68]. The SYBR green assay has high throughput and therefore it is commonly used as a preliminary screen in antimalarial drug discovery [69]. The compounds were initially screened at 1 µM and 10 µM concentrations together with chloroquine (CQ) and WR99210 controls (Figure S4, Supplementary information file 1). Out of the six compounds, **1** (CEP-37440) and **2 (**AZD-1480), exhibited promising antiplasmodial activity at the selected concentrations. Subsequently, IC_50_ was determined to be 1.22 µM and 4.00 µM for compounds **1** and **2**, respectively (Fig. 8; Table 4). Notably, all hits except compound 5, also received MAIP score higher than the average score of 56.32. Specifically, compounds **1** and **2** received the highest (101.68) and third-highest (76.16) scores among the six compounds, showing agreement between RF-1 and MAIP.

**Table 4:**
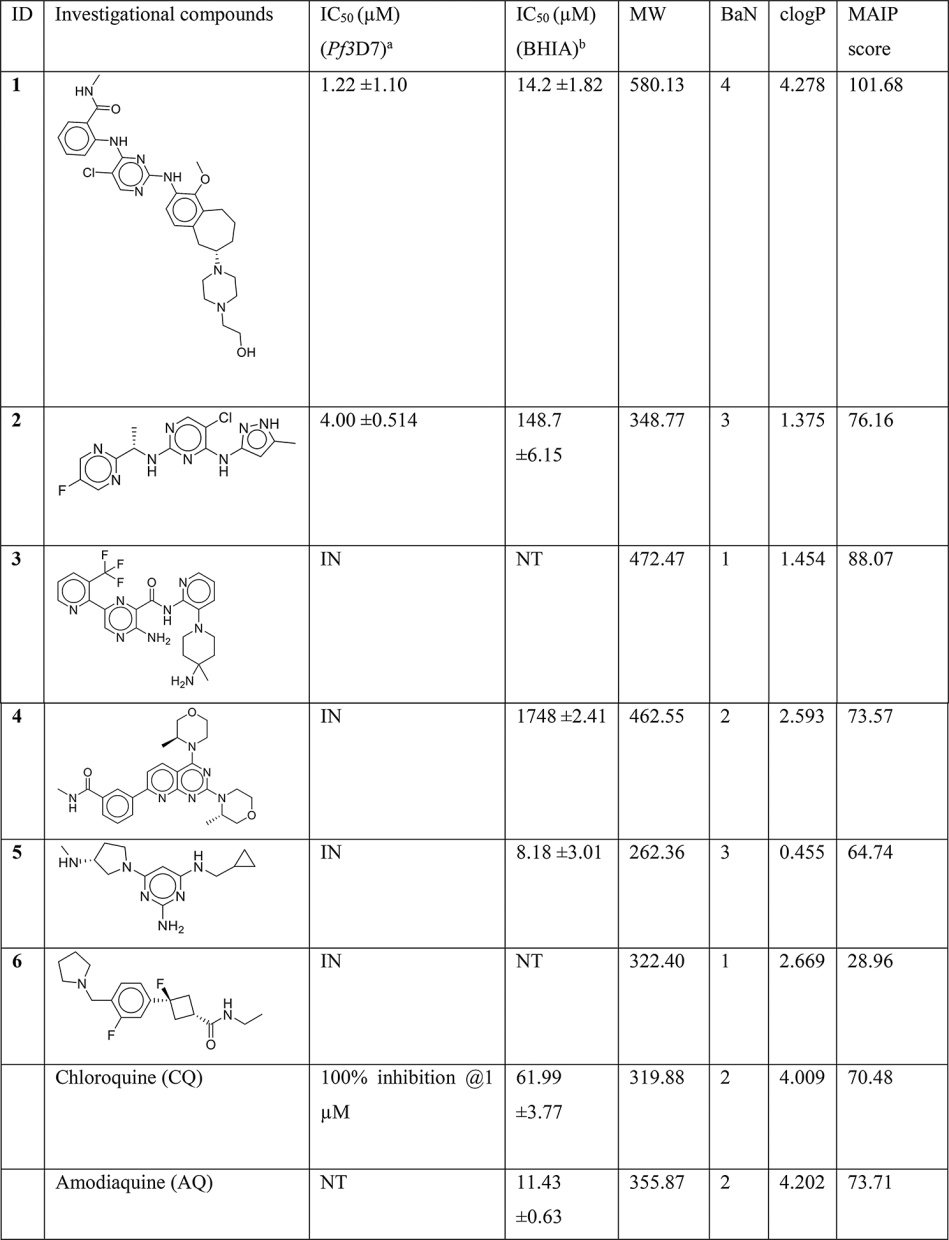
Structures, antiplasmodial activity, *β*-hematin inhibition activity, and physicochemical properties (predicted by Datawarrior [58]) of the purchased investigational compounds

^a^The values represent Mean ± SD from two biological replicates, each having three technical replicates

^b^The values represent Mean ± SD from three independent experiments, each having three replicates

IN = inactive; NT = Not tested

**Fig. 9:**
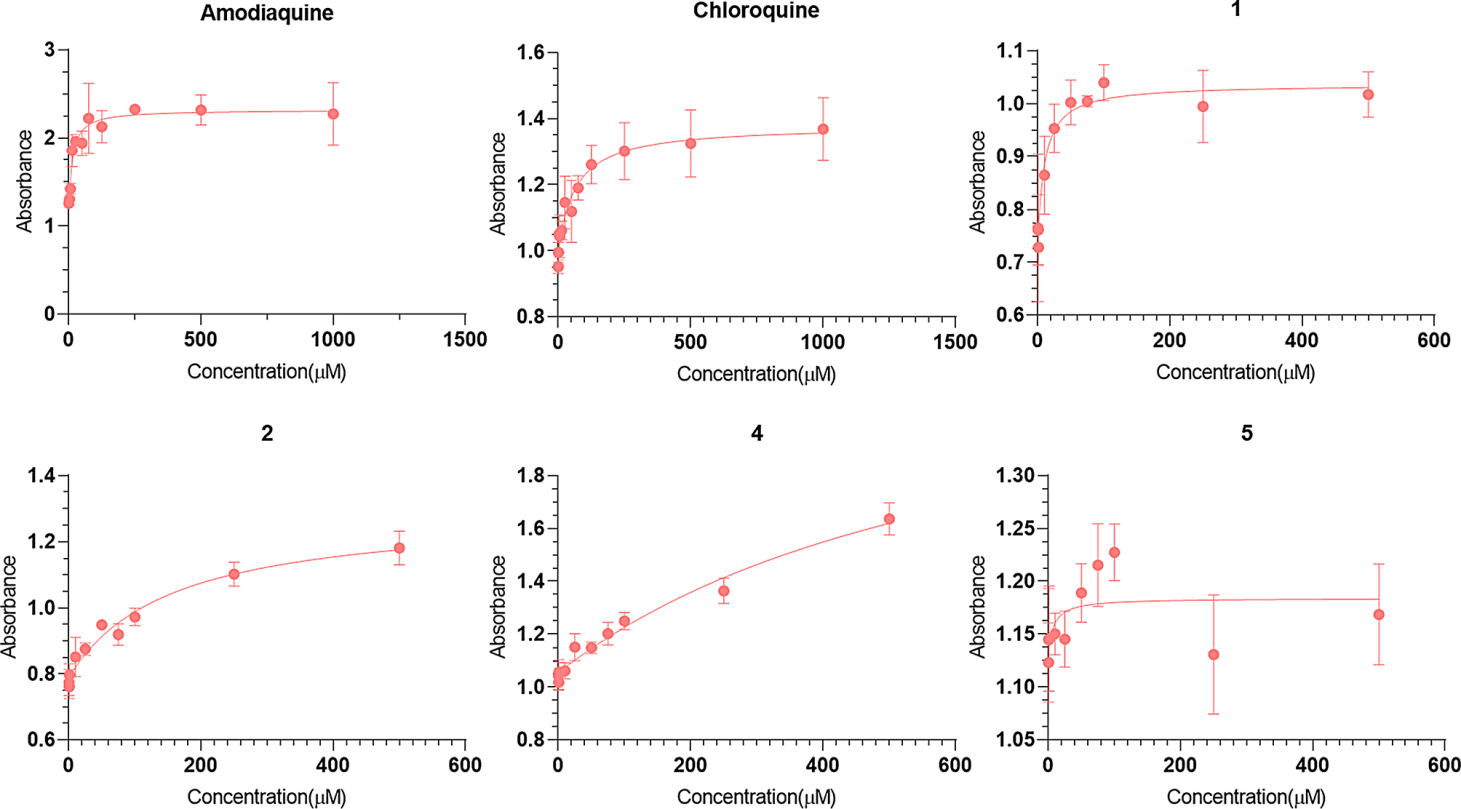
The dose response curves for the *β*-hematin formation inhibition of positive controls (amodiaquine and chloroquine) and the purchased compounds

Interestingly, both hit compounds (**1** and **2**) are human kinase inhibitors and have 2,4-diamino-5-chloropyrimidine chemotype. Compound **1** is a dual inhibitor of anaplastic lymphoma kinase (Alk) and focal adhesion kinase (Fak) and currently being evaluated in phase 1 clinical trials for antineoplastic activity. Compound **2** is the Janus-associated kinase 2 (JAK2) inhibitor, and it has been studied in phase 1 trials for the treatment of essential thrombocythaemia myelofibrosis, solid malignancies, post-polycythaemia vera, and primary myelofibrosis (Table S3, Supplementary information file 1). To investigate whether the homologue proteins are present in *Plasmodium* genome we performed the BLAST search using sequences of Alk (Uniprot ID Q9UM73), Fak (Uniprot ID Q05397), and JAK2 (Uniprot ID O60674). The search reveals the presence of several known and putative related kinases in different species of *Plasmodium* corresponding to Alk, Fak, and JAK2. The representative examples of the protein sequences from *P. falciparum* are shown in supplementary information (Figure S12, Supplementary information file 1). These findings suggest that the *Plasmodium* indeed has homologues of Alk, Fak, and JAK2 human targets and these parasite proteins provide opportunity for the design of novel antimalarial drug design. For instance, protein structures of the malarial homologues can be predicted and can be employed for structure-based design for the identification of new hits. It is highly plausible that compounds **1** and **2** might be targeting these *Plasmodium* kinases and therefore, these hits can be further optimized using the modeling studies. Indeed, several *Plasmodium* kinases play important roles at distinct stages of the malaria parasite life cycle and have been validated for antimalarial drug discovery [70–72]. Many case studies also demonstrate that it is possible to achieve human vs. *Plasmodium* selectivity while optimizing kinase inhibitors with potent in vitro and in vivo antimalarial activity [70, 73–76], which could be explored with these compounds.

In both hit molecules, substituents at position 2 and 4 of the chloropyrimidine ring are quite different and hence, these are considered diverse when evaluated by the Skelsphere descriptors. Searches in ChEMBL revealed that molecules similar (≥ 0.8) to **1** and **2** are reported in medicinal chemistry literature, but none have been evaluated against *P. falciparum*. Nonetheless, 2,4-diamino-pyrimidines with distinct substituents have been reported to possess antiplasmodial activity [18, 77, 78].

Four BaN centers are predicted in compound **1** as opposed to three BaNs in compound **2**. Moreover, compound **1** is expected to be more basic than compound **2** owing to the presence of piperazine ring bearing two tertiary amines that are absent in compound **2**. Also, compound **1** is significantly bulky (MW 580.13 Da) and more lipophilic (clogP 4.278) than compound **2** (MW 348.77; clogP 1.376). These, property differences are in line with the proposed antimalarial property space and suggest the possibility of hemozoin synthesis inhibition by **1** and **2** [23, 24]. Therefore, we screened all molecules, except **3** and **6** (due to insufficient quantity), in the *β*-hematin inhibition assay (BHIA) which is a commonly used surrogate of the Hz synthesis (Table 4; Fig. 9). Interestingly, compound **1** displayed inhibition in BHIA equipotent to the positive control amodiaquine (AQ) and ~ 4.4-fold potent than CQ. This observation suggests that Hz synthesis might be one of the targets of compound **1**. In contrast, compound **2** was observed to be ~ 10-fold and ~ 2.4-fold less potent than AQ and CQ, respectively, suggesting it to be a weak inhibitor of Hz synthesis. Compound **4** exhibited high IC_50_ in BHIA in accordance with its inactivity against the parasite. However, compound **5** was found to be the most potent inhibitor of *β*-hematin synthesis with IC_50_ 8.18 µM. The poor parasiticidal activity of compound **5** suggests that it might be unable to accumulate in DV of the parasite. Also, compound **5** is considerably small (MW 262.36) and less lipophilic (clogP 0.455) than **1** which doesn’t align with the ideal antimalarial property space. Particularly, the low lipophilicity might hinder compound **5** to access the Hz that grows at the lipid-water interface within the parasite’s DV.

## Comparison with artificial neural network (ANN) model

Further, we compared RF-1 with the modern ANN models, which are increasingly being applied in drug discovery [79–83]. In ANN, neurons represent basic units that are organized into several layers with specific roles. Thus, an interconnected network of input, hidden, and output layers is organized to build the ANN model. The input layer is the first layer to which features of the dataset are fed in numerical form. The hidden layers perform computations, adding weights and non-linear activation functions to the input numbers to achieve the desirable output during training. The output from one hidden layer may act as the input for the next hidden layer, thus increasing the complexity or ‘deepness’ of the model. The output layer produces the final prediction based on its own set of weights.

We employed a simple multi-layer perceptron (MLP) model for benchmarking. For training and validation of MLP, the same set of molecules and MFPs (Avalon) were used as in the case of RF-1. It is known that one to two hidden layers are sufficient for most of the predictive tasks [84, 85]. Also, it is proposed that the number of neurons in the hidden layer should be between the number of neurons in the input and the output layers [84], which is 1024 (MFP bits) and 2 (two classes), respectively, in this case. Hence, we initially employed a single hidden layer and varied the neuron numbers (25 to 250) and α values (0.1, 0.01, and 0.001). The MLP-8 model with a neuron size of 100 and alpha value of 0.001 was found to be optimum with 90.3% accuracy for the validation test set (Table 5). The neuron size higher than 100 resulted in slightly poor performance by the single-layer model. Further increasing the hidden layer size to two did not improve the accuracy (Table S4, Supplementary information file 1) hence, we used MLP-8 for further comparison. Ten-fold cross-validation demonstrated MLP-8 to be stable with reproducible values for accuracy and other parameters (Table S5, Supplementary information file 1).

**Table 5:** MLP models with a single hidden layer with varying neuron numbers

	Neuron numbers	Alpha	Accuracy (validation test set)
MLP-1	25	0.01	0.886
MLP-2	25	0.001	0.890
MLP-3	25	0.0001	0.891
MLP-4	50	0.01	0.876
MLP-5	50	0.001	0.896
MLP-6	50	0.0001	0.889
MLP-7	100	0.01	0.898
MLP-8	100	0.001	0.903
MLP-9	100	0.0001	0.901
MLP-10	150	0.01	0.897
MLP-11	150	0.001	0.891
MLP-12	150	0.0001	0.899
MLP-13	200	0.01	0.902
MLP-14	200	0.001	0.887
MLP-15	200	0.0001	0.898
MLP-16	250	0.01	0.899
MLP-17	250	0.001	0.888
MLP-18	250	0.0001	0.899

**Fig. 10:**
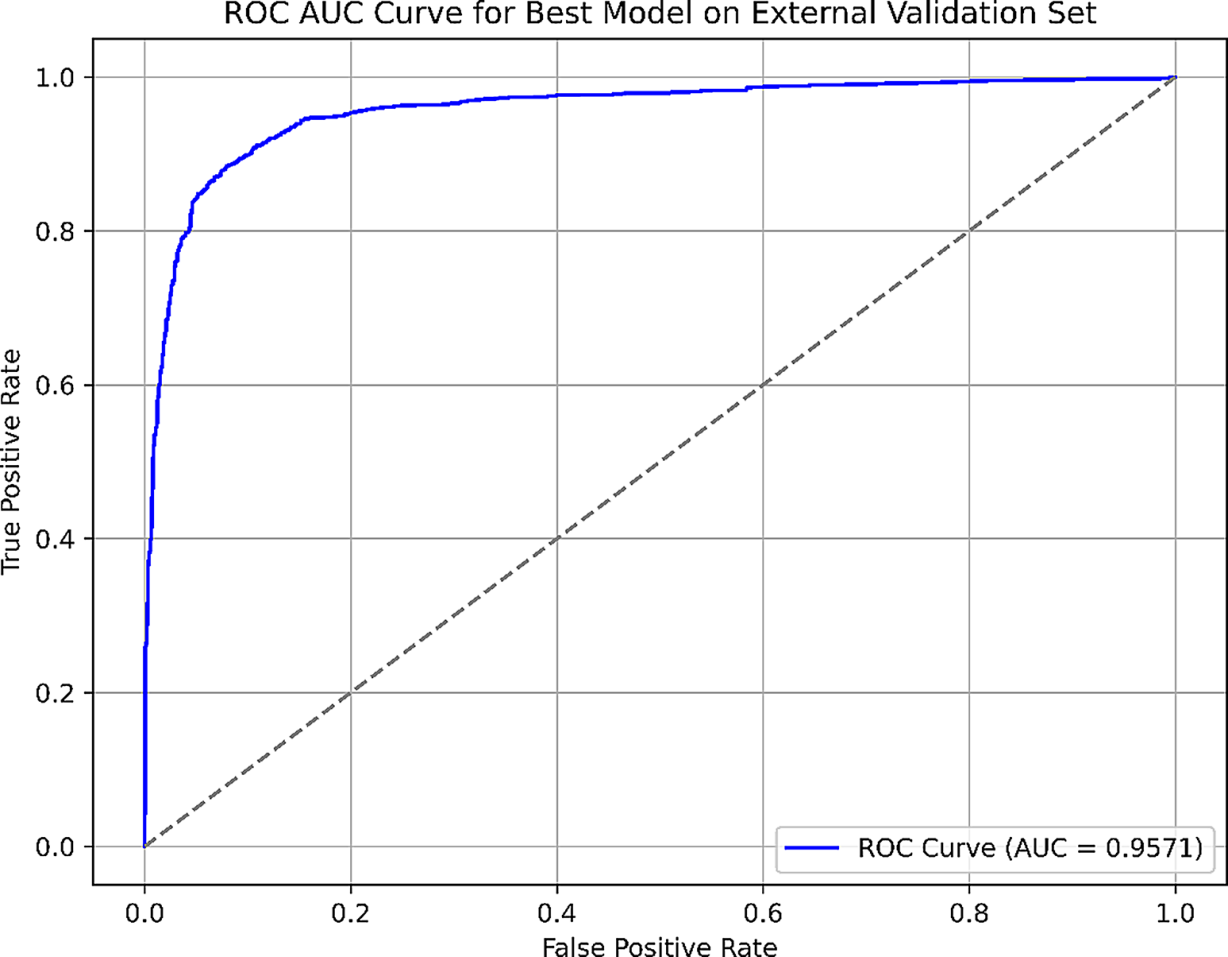
ROC curve obtained with MLP-8. The diagonal represents a random classifier with 50% AUROC

For the prediction of the external test set MLP-8 was found to be slightly less accurate than RF-1 (90.2% vs. 91.7%, Table 6). In addition, RF-1 performed equally well in terms of recall rates (88.4% vs. 88.9%) and slightly better in terms of precision (93.5% vs. 89.8%) and F-score (90.8% vs. 89.4%) (Table 6). Also, RF-1 was found to yield a higher value for AUROC (Fig. 10) compared to MLP-8 (97.3% vs. 95.7%).

**Table 6:** The comparison of prediction performance of models MLP-8 and RF-1 for the external test set

Evaluation metric	MLP-8	RF-1
Accuracy	0.902	0.917
Precision	0.898	0.935
Recall	0.889	0.884
F measure	0.894	0.908
AUROC	0.957	0.973

Overall, RF-1 seems to perform better than the MLP model for the used antiplasmodial datasets. Nevertheless, the difference between the two models is marginal, and further tuning and optimization of MLP might improve its performance.

## Conclusion

This work reports the training and validation of a robust RF model generated from a large, refined set of antimalarial molecules deposited in public repository ChEMBL. As opposed to the previously reported ML models which rely on HTS screening and/or proprietary data, RF-1 is based on the more dependable multi-dose activity. Among various tested MFPs, Avalon MFP yielded a model with the highest accuracy requiring the lowest number for trees and tree depth. The model was validated and evaluated through a variety of matrices and found to have high predictive performance for an external test set. The high accuracy, sensitivity, specificity, and AUROC of RF-1 ensures that it can be reliably used to distinguish between active and inactive antimalarial molecules. Thus, RF-1 can be employed for high throughput virtual screening (HTVS) using large chemical libraries making it possible to explore a wider chemical space. The molecules can be prioritized for experimental screening, reducing the time and cost involved in early drug discovery phase. This is particularly important in the case of antimalarial drug discovery since most of the affected population reside in low- and mid-income countries.

RF-1 was also found to be comparable to a recently reported MAIP SVM model in terms of the property space of the hits. Nonetheless, the hits predicted by RF-1 and MAIP from a common commercial library do not overlap reflecting different training sets and algorithms used to develop these models. Experimental validation of RF-1 was carried out by repurposing study with investigational agents. Two human kinase inhibitors (**1** and **2**) in clinical trials predicted to be active by RF-1 were found to possess low micromolar antiplasmodial and *β*-hematin inhibition activity. Thus, these hits represent excellent starting points for developing antimalarials for targeting both, the corresponding parasite kinases, and *β*-hematin. Such dual targeting antimalarials are expected to be less susceptible to resistance development by the parasite.

One major limitation of the work is that the dataset of antimalarials is obtained from ChEMBL which collects molecules from a selected set of medicinal chemistry journals. This limited dataset might not represent the complete structural diversity of antimalarials, thus affecting the accuracy and applicability of the model. In future, the training data can be updated from other sources to further improve the size and chemical diversity that might result in a model with a wider APD.

Overall, the RF model disclosed in this manuscript is a useful tool for identifying new antimalarial compounds. Given the complementarity of RF-1 with MAIP both can be used in consensus to select hits through HTVS. One important aspect of this study is the use of open-source data and KNIME workflow enabling usage by medicinal chemists with no coding expertise. This workflow can be used to generate models applicable to other targets since the basic steps of model building and validation remain the same. Importantly, this KNIME workflow can also be modified by replacing RF with other ML algorithms such as SVM, k-NN, NB, and XGBoost.

## Experimental

### Data curation and model building

Detailed methodology for the data collection is reported in our earlier works [23, 24]. Briefly, ChEMBL [32, 33](version 30) was searched within the Osiris Datawarrior program (v 5.50) [58] for molecules tested against *P. falciparum*. The small molecules within MW ≤ 900 Da with reported IC_50_/EC_50_ values were retained. The duplicate molecules were merged resulting in a total of 15,118 molecules. The average of IC_50_ values of the merged molecules was considered for defining the active (IC_50_ ≤ 200 nM) and inactive categories (IC_50 ≥_ 5000 nM).

KNIME software (v 5.1) [42–44] was used for developing the ML workflow using inbuilt and RDKit [46] community nodes. The molecules were standardized using the ‘RDkit from molecule’ converter node followed by the generation of different MFPs (1024 bits) using the ‘RDKit Fingerprint’ node. The MFP bit vectors were then split into individual integer columns using the ‘Expand Bit Vector’ node. 20% of the dataset was split into the external test set (*N* = 3024; Supplementary information file 4) used to evaluate the final model employing the stratified sampling. The remaining molecules were further split into training (75%, *N* = 9070; Supplementary information file 2) and internal validation set (25%, *N* = 3024; Supplementary information file 3). The latter was used to validate the hyperparameters of the ‘RF Learner’ node. During optimization, nT varied from 50 to 400 while Td varied between 10 and 50, with the step size of 50 and 10, respectively. All the possible combinations of the nT and Td values were tested using the ‘brute force’ setting in the ‘Parameter Optimization Loop’ node. The training set data was fed to the ‘RF Learner’ node which utilizes the Classification And Regression Trees (CART) algorithm [86]. During RF model building the individual trees are constructed with *m*_try_ descriptors rather than the total number of *p* descriptors. In KNIME ‘RF Learner’ node, the *m*_try_ value is fixed to the square root of *p* which is shown to perform well [35]. The MFP expanded 1024-bit columns (e.g. Avalon) were selected as the attributes while the target class was set to the column with class description (active/inactive) for each training set molecule. The ‘Information Gain’ split criteria was employed for individual tree construction.

Thus, models were generated using the training set (*N* = 9070) using different MFPs and the values of nT and Td were optimized to obtain maximum accuracy for the internal validation set (*N* = 3024). The optimized models were finally evaluated for their predictive performance using the external test set (*N* = 3024). Various performance matrices were calculated using the ‘Scorer’ and ‘ROC curve’ nodes of KNIME. The confusion matrix and equations for calculating various performance matrices are provided in Figure S1 with Supplementary information file 1.

The applicability domain (APD) [52–54] of RF-1 was calculated using the ‘Domain similarity’ node from Enalos [87]. The distance of a test compound to its nearest neighbour in the training set was compared to the predefined applicability domain (APD) threshold. The process involves the calculation of average Euclidean distances (d) between all pairs of compounds in the training set and the corresponding standard deviation (σ). The APD threshold was then determined using the equation APD = ‘d’ + Zσ, where Z is an empirical cutoff of 0.5 [54]. Any molecule in the test set with APD higher than the APD threshold was considered to have an unreliable prediction.

The final Knime workflow is provided in the supplementary information (Supplementary information file 5).

### Testing antiplasmodial activity of select compounds using SYBR green assays

All investigational compounds were purchased from MedChemExpress (MCE^®^) in sufficient purity (> 98%). The characterization data was obtained from the vendor and is provided in Supplementary information file 1 (Figure S5 -S11). The 3D7 strain of *P. falciparum* was a kind gift from MR4, BEI Resources (NIAID, USA). ABS of 3D7 were grown in O + blood in RPMI complete medium using established protocols [88]. SYBR green-based ring stage killing assays were performed as previously described [68, 89]. 

### BHIA screening

The *β*-hematin inhibition activity of selected compounds was performed by applying the previously reported detergent-mediated NP-40 assay [90, 91]. The clinically used antimalarial drugs AQ (purity > 98%, Cat. No. A3133, TCI), and CQ (purity > 98%, Cat. No. C6628, Sigma-Aldrich) were taken as positive controls. The stock solution of 20 mM concentration was prepared for all the drugs in DMSO except for CQ which was dissolved in water. Further dilutions for all drugs were made using NP-40/water (61.1 mM). The stock solution of hematin was prepared by sonicating hemin chloride in DMSO. Subsequently, the stock solution of hemin chloride was suspended in 1 M acetate buffer (PH 4.8) followed by vortex. The drug (50 µL) and suspended hemin chloride solution (48 µL) were added to the 96 well plate and incubated at ambient temperature (37 ^o^C) for 6 h. The assay was analysed using the pyridine-ferrochrome method developed by Ncokazi and Egan. The pyridine solution (PH 7.4) was prepared by combining 50% (v/v) pyridine, 30% (v/v) water, and 20% (v/v) acetone and 2 M 4-(2hydroxyethyl)-1-piperazineethanesulfonic acid (HEPES). 32 µL of this solution was added to each well followed by 60 µL of acetone to assist the hematin dispersion. The absorbance of the plate was taken at 405 nm using Biotech Epoch Microplate Reader. The IC_50_ of each compound was calculated by plotting sigmoidal dose-response curves in GraphPad Prism v 8.0.0. (GraphPad Software Inc., La Jolla, CA, USA).

### MLP modeling

The MLP models were built using scikit-learn (v 1.5.0), a Python (3.11.9) based library. The training dataset was made of a matrix of size 9070 × 1024, where each row represents a single distinct molecule, and the columns represent their respective Avalon fingerprints as X (variable). The activity class (Active: 1, Inactive: 0) was represented by Y-label. Similarly, the two validation datasets (both internal and external) were of a matrix size of 3024 × 1024. Multiple MLP models were trained based on the range of hyperparameters provided using the training set (Table 5). The trained models were tested for their performance on internal validation and were ranked based on their accuracy parameter. The optimized model (MLP-8) was further evaluated against the external test dataset. The complete Python code used to develop the MLP models can be accessed at Github (https://github.com/sharma-lakshya/MLP_model_comparison).

## Electronic supplementary material

Below is the link to the electronic supplementary material.

Supplementary Material 1

Supplementary Material 2

Supplementary Material 3

Supplementary Material 4

Supplementary Material 5

## Data Availability

The datasets used in this study are available in either in the main manuscript or as supplementary material. The Supplementary Figures S1 – S12 and Tables S1 – S5 are available in the Supplementary Information file S1. The training set (Supplementary Information file 2), internal validation test set (Supplementary Information file 3), and external test set (Supplementary Information file 4) are provided as separate CSV files. The Knime workflow is provided as the Supplementary Information file 5 material. The Python code for the MLP modelling can be found at https://github.com/sharma-lakshya/MLP_model_comparison.
