# Supplementary material for: Development and experimental validation of a machine learning model for the prediction of new antimalarials
Source: BMC Chem. 2025 Jan 30;19(1):28. doi: 10.1186/s13065-025-01395-4 (PMC11783816; doi:10.1186/s13065-025-01395-4)
Supplement: Supplementary file 1 — Supplementary Material 1 [file 13065_2025_1395_MOESM1_ESM.docx]

Development and experimental validation of a machine learning model for the prediction of new antimalarials

Mukul Kore^a^, Dimple Acharya^b^, Lakshya Sharma^a^, Shruthi Sridhar Vembar^b^, Sandeep Sundriyal^a*^

^a^Department of Pharmacy, Birla Institute of Technology and Science Pilani, Pilani Campus, Vidya Vihar, Pilani, Rajasthan 333 031, India

^b^Institute of Bioinformatics and Applied Biotechnology, Helix Biotech Park, Electronics City Phase I, Bengaluru, Karnataka, 560100, India.

**^*^**Email: [sandeep.sundriyal@pilani.bits-pilani.ac.in](mailto:sandeep.sundriyal@pilani.bits-pilani.ac.in)

**Content**

**Supplementary figures**

**Fig. S1** Confusion matrix for external test set 3

**Fig. S2** Structure of molecules obtained from model 1 having 70% similarity with MAIP model 4

**Fig. S3** Structure of the common hits obtained from Model 1 and MAIP model 5

**Fig. S4** Preliminary antiplasmodial activity of compounds **1**-**6** 6

**Fig. S5** Spectral data of compound **1** 7

**Fig. S6** Spectral data of compound **2** 8

**Fig. S7** Spectral data of compound **2** 9

**Fig. S8** Spectral data of compound **3** 10

**Fig. S9** Spectral data of compound **4** 11

**Fig. S10** Spectral data of compound **5** 12

**Fig. S11** Spectral data of compound **6**  13

**Fig. S12** BLAST search results 14

**Supplementary Tables**

**Table S1** The model performance data obtained from the OOB confusion matrix 15

**Table S2** Statistical details of external test set 15-16

**Table S3** Details of the investigational compounds 16-18

**Table S4** MLP models with two hidden layers 18-19

**Table S5** Ten-fold cross-validation of MLP-8 29-20


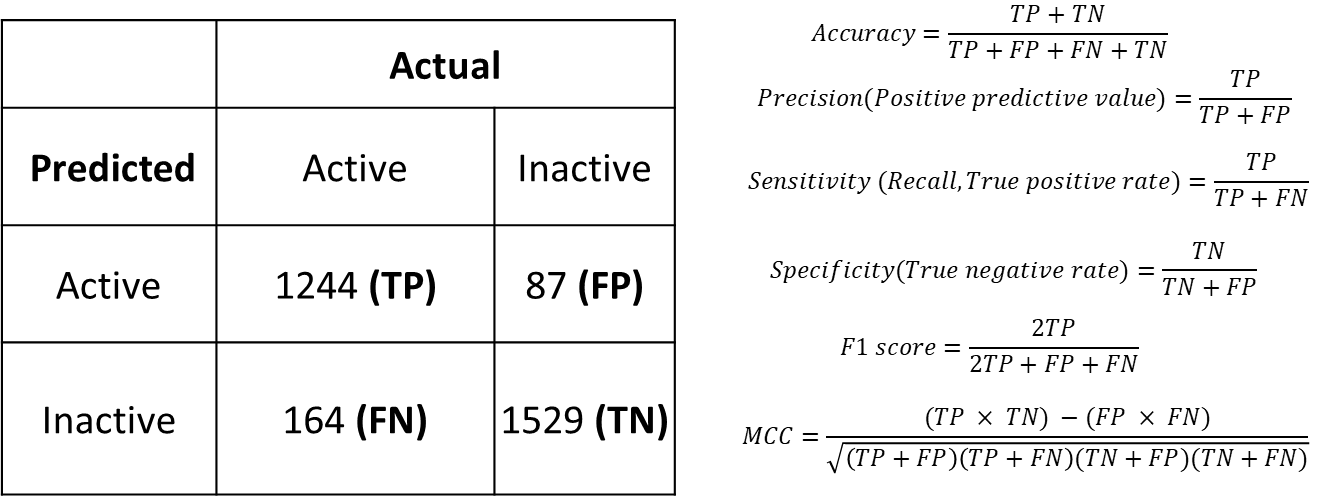


**Fig. S1** The confusion matrix obtained for the external test set using Model 1 and the equations for calculating various model performance matrices.





**Fig. S2** The 30 Enamine hits obtained from Model 1, possessing at least 70% similarity to one of the MAIP model hit. The top left corner represents the molecule ID while bottom left corner represents the highest Skelsphere similarity.


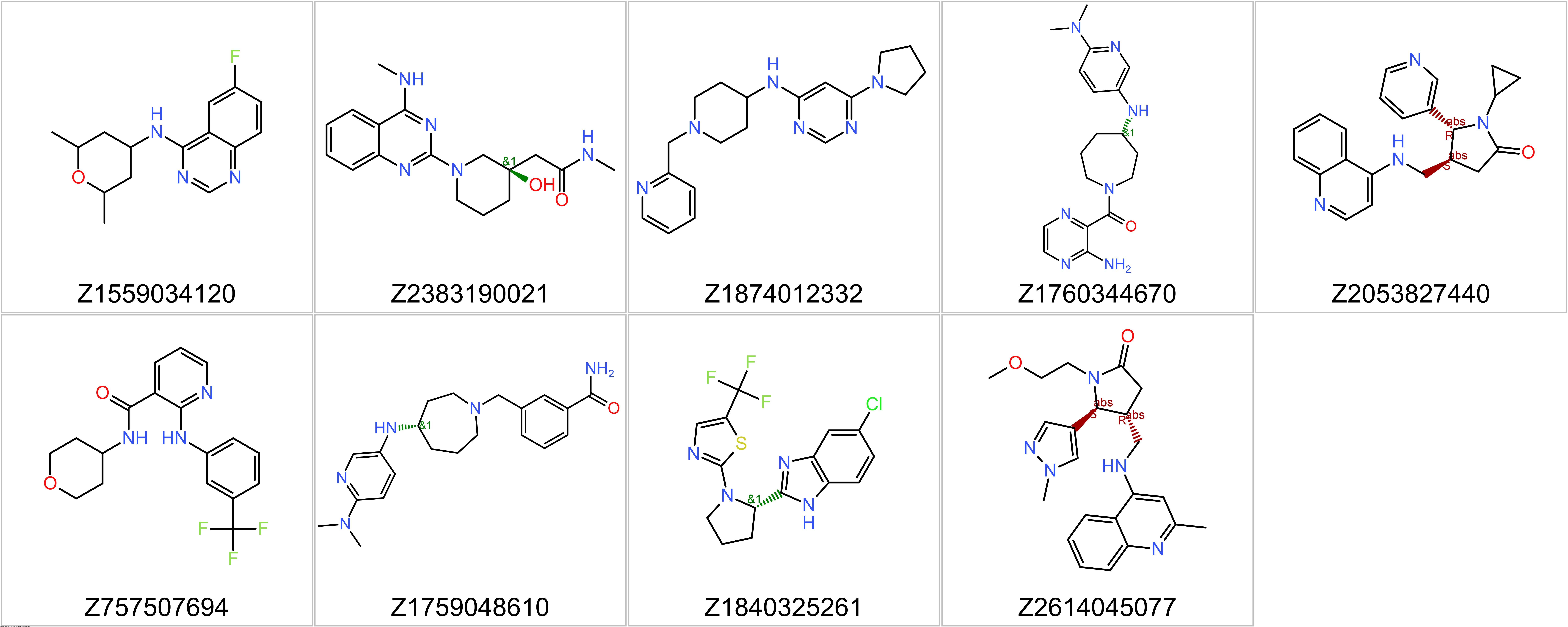


**Fig. S3** The 9 common hits obtained from Model-1 and MAIP model after screening the Enamine diversity set of ~ 10 K compounds.


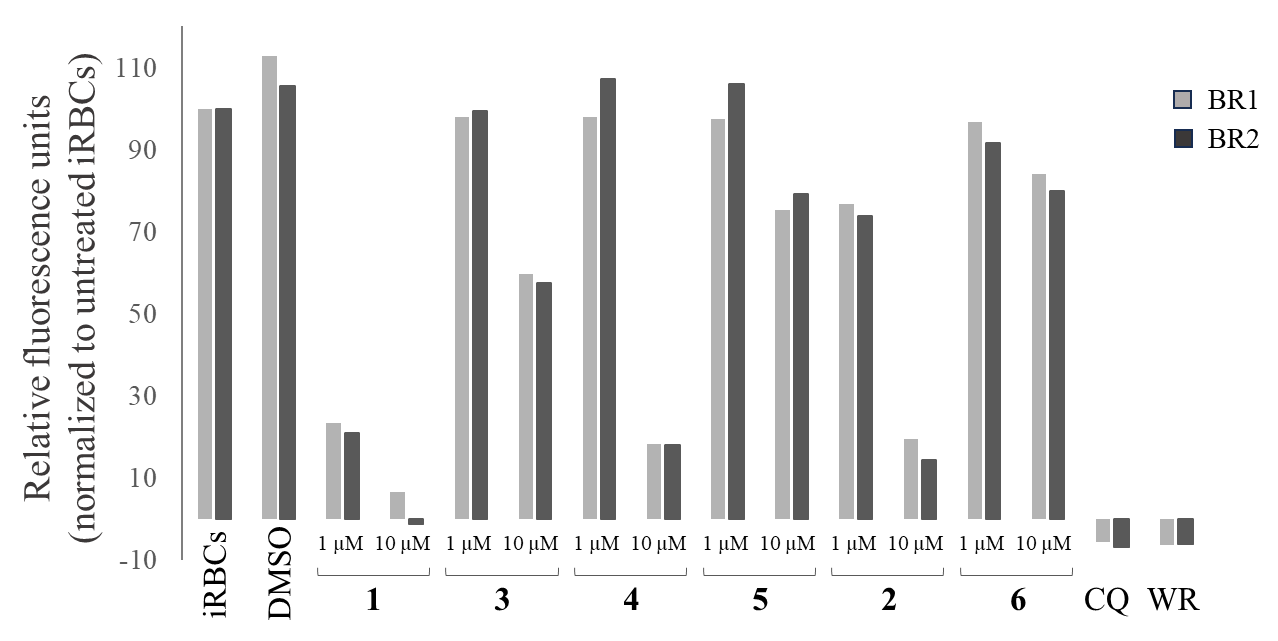


**Fig. S4** Preliminary antiplasmodial activity of compounds **1**-**6** at 1 µM and 10 µM. The known antimalarials chloroquine (CQ) and WR99210 were used as positive controls.

Characterization data of the purchased compounds **1 – 6** obtained from the vendor


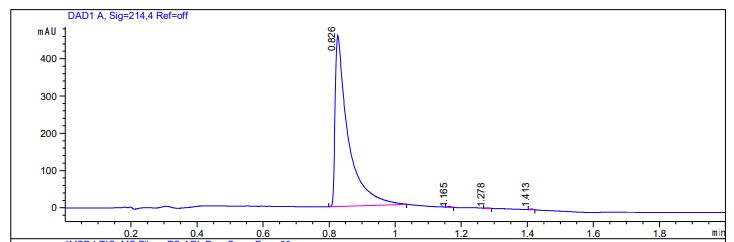


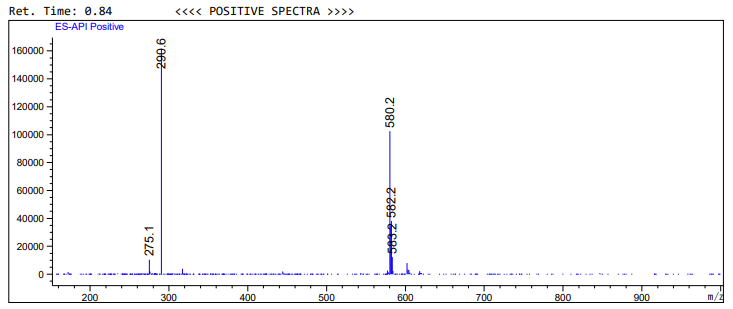


**Fig. S5** LCMS spectral data of compound **1**


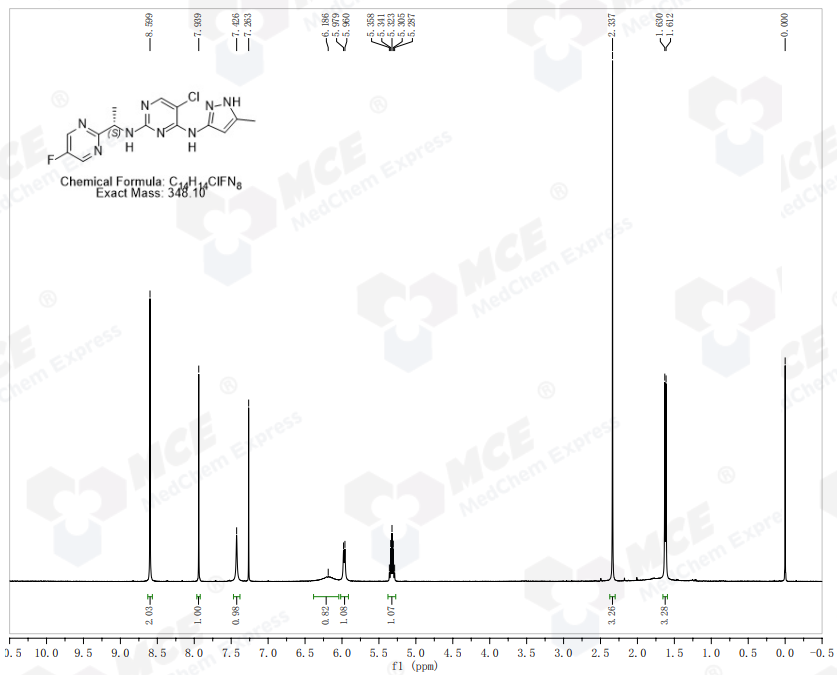


**Fig. S6** ^1^H NMR spectral data of compound **2**


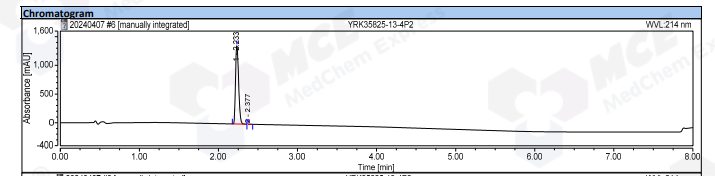


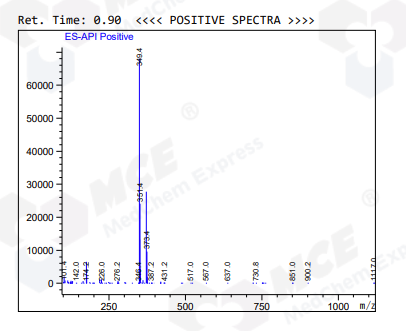


**Fig. S7** LCMS spectral data of compound **2**


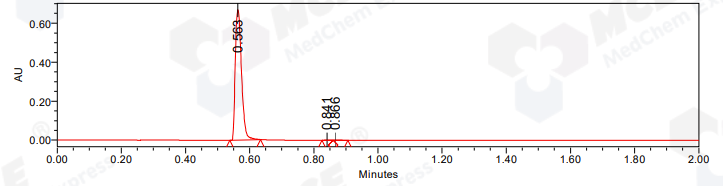


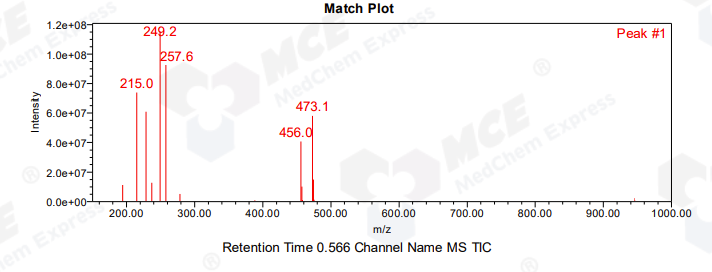


**Fig. S8** LCMS spectral data of compound **3**


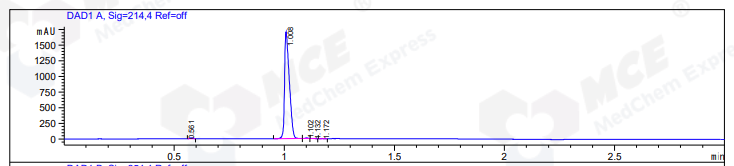


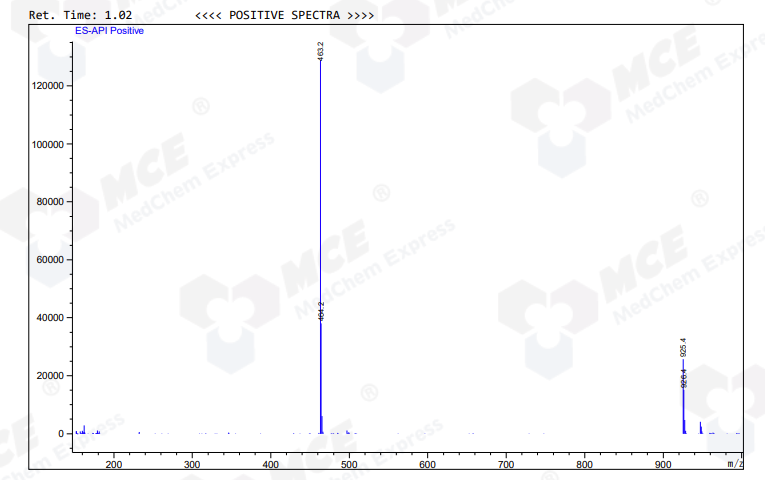


**Fig. S9** LCMS spectral data of compound **4**


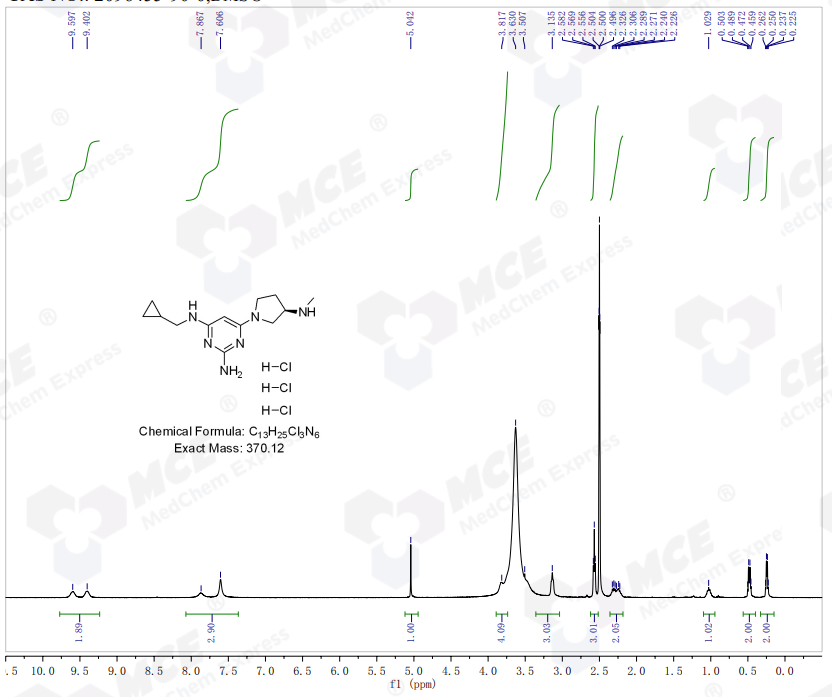


**Fig. S10** ^1^H NMR spectral data of compound **5**


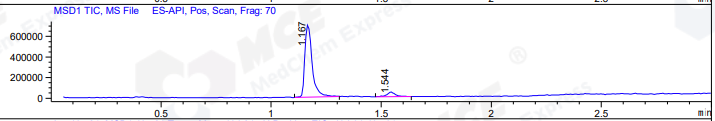


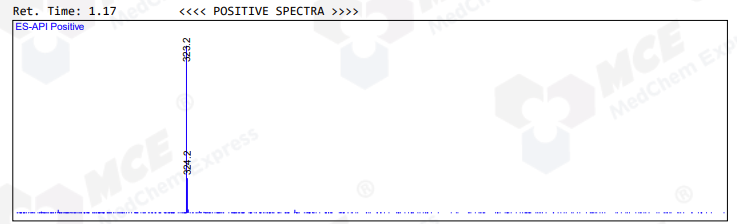


**Fig. S11** LCMS spectral data of compound **6**

**A: AUR protein kinase [*Plasmodium falciparum* UGT5.1]; Sequence ID: EWC78760.1; Length: 952**


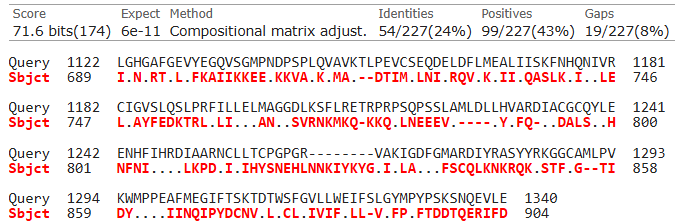


**B: TKL protein kinase [*Plasmodium falciparum* Tanzania (2000708)]; Sequence ID: ETW38949.1; Length: 962**


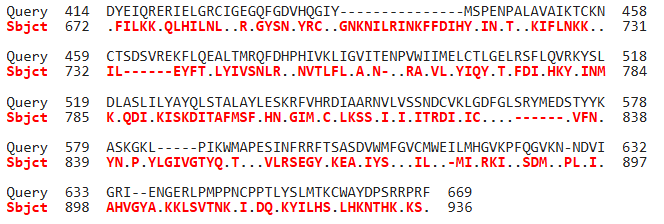


**C: Serine/threonine protein kinase [*Plasmodium falciparum* UGT5.1]; Sequence ID: EWC77408.1; Length: 224**

**
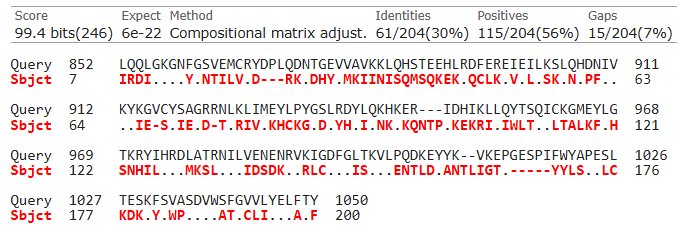
**

**Fig. S12**. BLAST search results in *Plasmodium* (taxid:5820) using queries A) Alk (Uniprot ID Q9UM73) B) Fak (Uniprot ID Q05397), and C) JAK2 (Uniprot ID O60674). Several similar proteins are found in different species of *Plasmodium*. The matching protein sequence with the highest score in *P. falciparum* are shown as the representative examples, for each query.

**Table S1** The model performance matrices obtained from the OOB confusion matrix using optimized values of RF hyperparameters. (nT = 100; Td = 50). The values are close to the performance of Model 1 for test set confusion matrix (see Table 1 of the manuscript).

| TP | FP | TN | FN | Recall | Precision | Sensitivity | Specificity | F-measure | Accuracy | Cohen's kappa |
| --- | --- | --- | --- | --- | --- | --- | --- | --- | --- | --- |
| 3696 | 288 | 4559 | 527 | 0.875 | 0.928 | 0.875 | 0.941 | 0.901 |  |  |
| 4559 | 527 | 3696 | 288 | 0.941 | 0.896 | 0.941 | 0.875 | 0.918 |  |  |
|  |  |  |  |  |  |  |  |  | 0.910 | 0.819 |

**Table S2** Statistical details associated with Figure 6 of the manuscript. The p-value < 0.00005 is rounded off to 0.

| **MW** |  |  |  |  |  |  |  |  |  |  |  |
| --- | --- | --- | --- | --- | --- | --- | --- | --- | --- | --- | --- |
| Prediction | Total | Outliers | Mean | 1st Quart. | Median | 3rd Quart. | Lower Adj. Lt. | Upper Adj. Lt. | Std. Dev. | Conf. Intv. (95%) | p-Value |
| Active (ref) | 129 | 8 | 343.7 | 327.17 | 343.39 | 366.7 | 275.31 | 418.92 | 38.325 | 337.09-350.31 |  |
| Inactive | 10111 | 215 | 331.2 | 309.41 | 332.47 | 353.31 | 244.25 | 418.92 | 35.927 | 330.54-331.94 | 0.00035 |
|  |  |  |  |  |  |  |  |  |  |  |  |
| **clogP** |  |  |  |  |  |  |  |  |  |  |  |
| Prediction | Total | Outliers | Mean | 1st Quart. | Median | 3rd Quart. | Lower Adj. Lt. | Upper Adj. Lt. | Std. Dev. | Conf. Intv. (95%) |  |
| Active | 129 | 1 | 1.754 | 0.97525 | 1.7611 | 2.4556 | -0.3955 | 4.3177 | 1.024 | 1.5767-1.9302 |  |
| Inactive | 10111 | 58 | 1.283 | 0.48333 | 1.2761 | 2.0577 | -1.8767 | 4.4152 | 1.1463 | 1.2606-1.3053 | 0 |
|  |  |  |  |  |  |  |  |  |  |  |  |
| **TPSA** |  |  |  |  |  |  |  |  |  |  |  |
| Prediction | Total | Outliers | Mean | 1st Quart. | Median | 3rd Quart. | Lower Adj. Lt. | Upper Adj. Lt. | Std. Dev. | Conf. Intv. (95%) |  |
| Active | 129 | 0 | 74.73 | 62.21 | 73.05 | 88.618 | 37.39 | 120.32 | 17.689 | 71.676-77.782 |  |
| Inactive | 10111 | 136 | 79.45 | 66.93 | 79.7 | 91.73 | 32.26 | 128.9 | 18.852 | 79.087-79.822 | 0.0031 |
|  |  |  |  |  |  |  |  |  |  |  |  |
| **#BaN** |  |  |  |  |  |  |  |  |  |  |  |
| Prediction | Total | Outliers | Mean | 1st Quart. | Median | 3rd Quart. | Lower Adj. Lt. | Upper Adj. Lt. | Std. Dev. | Conf. Intv. (95%) |  |
| Active | 129 | 3 | 0.798 | 0 | 1 | 1 | 0 | 2 | 0.78442 | 0.66308-0.93382 |  |
| Inactive | 10111 | 75 | 0.445 | 0 | 0 | 1 | 0 | 2 | 0.63214 | 0.43313-0.45778 | 0 |
|  |  |  |  |  |  |  |  |  |  |  |  |
| **#AR** |  |  |  |  |  |  |  |  |  |  |  |
| Prediction | Total | Outliers | Mean | 1st Quart. | Median | 3rd Quart. | Lower Adj. Lt. | Upper Adj. Lt. | Std. Dev. | Conf. Intv. (95%) |  |
| Active | 129 | 50 | 2.031 | 2 | 2 | 2 | 2 | 2 | 0.62422 | 1.9233-2.1387 |  |
| Inactive | 10111 | 48 | 1.735 | 1 | 2 | 2 | 0 | 3 | 0.7185 | 1.7214-1.7494 | 0 |
|  |  |  |  |  |  |  |  |  |  |  |  |
| **MAIP Score** |  |  |  |  |  |  |  |  |  |  |  |
| Prediction | Total | Outliers | Mean | 1st Quart. | Median | 3rd Quart. | Lower Adj. Lt. | Upper Adj. Lt. | Std. Dev. | Conf. Intv. (95%) |  |
| Active | 129 | 0 | 30.03 | 21.916 | 29.835 | 37.843 | 9.289 | 55.174 | 10.578 | 28.202-31.852 |  |
| Inactive | 10111 | 79 | 19.6 | 12.335 | 19.242 | 26.356 | -8.3398 | 47.343 | 10.508 | 19.393-19.802 | 0 |

**Table S3** Details of the investigational compounds purchased from MedChemExpress (MCE^®^) screened against *P. falciparum*.

| ID | Investigational compounds | Target (Indication) | Catalogue No. and purity |
| --- | --- | --- | --- |
| **1** |   CEP-37440 (Phase1)  Pubchem ID 71721648 | *Anaplastic lymphoma kinase (Alk) and focal adhesion kinase (Fak) dual inhibitor*  Antineoplastic agent | Cat. No.: HY-15841  Purity: 99.6% |
| **2** |   AZD1480 (Phase 1)  Pubchem ID  16659841 | *Janus-associated kinase 2 (JAK2) inhibitor*  Essential thrombocythemia myelofibrosis / Post molycythemia vera Myelofibrosis / Primary myelofibrosis (PMF) | Cat. No.: HY-10193  Purity: 99.9% |
| **3** |   Darovasertib/LXS196  Phase 2/Phase 3  Pubchem ID 118873253 | *Protein kinase C inhibitor* | Cat. No.: HY-101569  Purity: 99.4% |
| **4** |   Vistusertib/AZD-2014  Phase 1/Phase 2  Pubchem ID 25262792 | *Mammalian target of rapamycin (mTOR) inhibitor*  Under investigation for the treatment of Advanced Gastric Adenocarcinoma | Cat. No.: HY-15247  Purity: 98.9% |
| **5** |   Adriforant/PF-3893787  Phase 2 (Terminated)  Pubchem ID 24745335 | *Histamine H4 receptor antagonist*  Investigated as anti-inflammatory agent in patients with plaque psoriasis | Cat. No.: HY-19705B  Purity: ≥98.0% |
| **6** |   PF-03654746  Phase 1/2  Pubchem ID 16119086 | *Human H3 receptor antagonist*  Being investigated for attention deficit hyperactivity disorder (ADHD) | Cat. No.: HY-11044  Purity: ≥99.0% |

**Table S4** MLP models with two hidden layers and varying neuron numbers.

| Neuron numbers | alpha | Accuracy (validation test set) |
| --- | --- | --- |
| (25, 25) | 0.01 | 0.887 |
| (25, 25) | 0.001 | 0.892 |
| (25, 25) | 0.0001 | 0.886 |
| (50, 50) | 0.01 | 0.894 |
| (50, 50) | 0.001 | 0.890 |
| (50, 50) | 0.0001 | 0.895 |
| (75, 75) | 0.01 | 0.889 |
| (75, 75) | 0.001 | 0.888 |
| (75, 75) | 0.0001 | 0.883 |
| (100, 100) | 0.01 | 0.900 |
| (100, 100) | 0.001 | 0.896 |
| (100, 100) | 0.0001 | 0.894 |
| (150, 150) | 0.01 | 0.892 |
| (150, 150) | 0.001 | 0.897 |
| (150, 150) | 0.0001 | 0.897 |
| (200, 200) | 0.01 | 0.896 |
| (200, 200) | 0.001 | 0.896 |
| (200, 200) | 0.0001 | 0.897 |
| (250, 250) | 0.01 | 0.887 |
| (250, 250) | 0.001 | 0.895 |
| (250, 250) | 0.0001 | 0.901 |

**Table S5** Ten-fold cross-validation of MLP-8

| Seed | Accuracy | Precision | Recall | Specificity | Cohen’s Kappa | F-measure | AUROC |
| --- | --- | --- | --- | --- | --- | --- | --- |
| 0 | 0.900 ± 0.013 | 0.895 ± 0.032 | 0.891 ± 0.018 | 0.908 ± 0.033 | 0.800 ± 0.026 | 0.893 ± 0.013 | 0.956 ± 0.007 |
| 1 | 0.905 ± 0.006 | 0.904 ± 0.016 | 0.890 ± 0.024 | 0.918 ± 0.016 | 0.808 ± 0.013 | 0.897 ± 0.007 | 0.958 ± 0.003 |
| 2 | 0.904 ± 0.008 | 0.910 ± 0.019 | 0.881 ± 0.023 | 0.924 ± 0.016 | 0.806 ± 0.017 | 0.895 ± 0.010 | 0.957 ± 0.007 |
| 3 | 0.903 ± 0.009 | 0.900 ± 0.022 | 0.890 ± 0.015 | 0.914 ± 0.017 | 0.804 ± 0.018 | 0.895 ± 0.01 | 0.958 ± 0.005 |
| 4 | 0.904 ± 0.008 | 0.893 ± 0.020 | 0.902 ± 0.023 | 0.905 ± 0.019 | 0.806 ± 0.016 | 0.897 ± 0.010 | 0.959 ± 0.008 |
| 5 | 0.902 ± 0.006 | 0.902 ± 0.018 | 0.888 ± 0.019 | 0.915 ± 0.016 | 0.804 ± 0.011 | 0.894 ± 0.007 | 0.959 ± 0.004 |
| 6 | 0.906 ± 0.005 | 0.905 ± 0.012 | 0.892 ± 0.017 | 0.919 ± 0.012 | 0.811 ± 0.011 | 0.898 ± 0.008 | 0.958 ± 0.007 |
| 7 | 0.906 ± 0.007 | 0.905 ± 0.019 | 0.893 ± 0.019 | 0.918 ± 0.017 | 0.812 ± 0.014 | 0.899 ± 0.007 | 0.958 ± 0.004 |
| 8 | 0.907 ± 0.007 | 0.906 ± 0.017 | 0.893 ± 0.018 | 0.919 ± 0.016 | 0.813 ± 0.014 | 0.899 ± 0.009 | 0.960 ± 0.006 |
| 9 | 0.906 ± 0.009 | 0.897 ± 0.010 | 0.903 ± 0.016 | 0.909 ± 0.009 | 0.812 ± 0.017 | 0.900 ± 0.01 | 0.959 ± 0.005 |
